# Supplementary material for: DNA double-strand break end synapsis by DNA loop extrusion
Source: Nat Commun. 2023 Apr 6;14:1913. doi: 10.1038/s41467-023-37583-w (PMC10079674; doi:10.1038/s41467-023-37583-w)
Supplement: Supplementary file 1 — Supplementary Information [file 41467_2023_37583_MOESM1_ESM.pdf]

Supplementary Information for:
DNA double-strand break end synapsis by DNA loop extrusion

Jin H. Yang[1,2,3], Hugo B. Brandão[1,2,3,4,*], and Anders S. Hansen[1,2,3,*]

[1] *Department of Biological Engineering, Massachusetts Institute of Technology, Cambridge, 02139*
[2] *The Broad Institute of MIT and Harvard, Cambridge, 02142*
[3] *Koch Institute for Integrative Cancer Research, Cambridge, 02142*
[4] *Present address: Illumina Inc., San Diego, 92122*
[*] *Corresponding authors: Hugo B. Brandão (articles@hugoresearch.com); Anders S. Hansen (ashansen@mit.edu)*

Contents

1 Supplementary Note 1 **2**
1.1 The probability of synapsis mediated by loop-extruding factors 2
1.2 The probability of DSB ends being constrained 3
1.2.1 Simplified expression for the probability of DSB ends being constrained 8
1.3 The probability of end joining given that DSB ends are constrained 8
1.3.1 The gap-bridging time distribution 8
1.3.1.1 The loading time distribution 8
1.3.1.2 The extrusion time distribution 9
1.3.1.3 Computing the gap-bridging time distribution through convolution 9
1.3.2 The constraining LEF lifetime distribution 10
1.3.3 The probability of gap-bridging on one side of the DSB 10
1.3.4 Simultaneous gap-bridging on both sides of the DSB and the gap-bridging LEF lifetime distribution 11
1.3.5 Computing the probability of joining DSB ends given DSB ends are constrained 12
1.3.6 Simplified expression for the probability of end-joining given the DSB is constrained and two important relative timescales 14
1.4 Synapsis with additional mechanisms 15
1.4.1 Synapsis with stabilized LEFs by BEs 15
1.4.2 Synapsis with a small fraction of long-lived LEFs 18
1.4.3 Synapsis with LEFs stabilized by DSB ends 20
1.4.4 Synapsis with targeted loading of LEFs to DSB ends 21
1.4.5 Synapsis with all four mechanisms combined 22
1.5 Simplified expression for the probability of synapsis with four additional mechanisms 24
1.6 Two important relative timescales underpinning synapsis efficiency 25
1.7 Limitations of our analytical theory and model 26
1.8 Conclusion 27

2 Supplementary Note 2 **28**
2.1 Estimation of the range of LEF separation and processivity 28
2.2 Estimation of the fold increase in LEF lifetime upon stabilization by BEs 29
2.3 Estimation of the fraction and lifetime of long-lived LEFs 29
2.4 Estimation of the fold increase in LEF lifetime upon stabilization by DSB ends 30
2.5 Estimation of the fold increase in LEF loading probability at DSB 30

3 Supplementary Figures **32**

1 Supplementary Note 1

Overview

To understand how the probability of DSB synapsis is affected by loop extruding factors (LEFs), we developed a probability theory framework for the process and used it to derive an analytical solution for the probability of synapsis. Briefly, we focus on the repair of DNA DSBs by non-homologous end joining (NHEJ). NHEJ repair involves two steps. First, the two DSB ends must be brought into proximity (synapsis). Second, they must be ligated back together. Here we focus on the first step, DSB synapsis. Please note that our goal is not to obtain the most precise analytical expression, but rather to derive a sufficiently accurate expression that we can use to obtain mechanistic intuition for how various loop extrusion mechanisms and parameters affect the efficiency of synapsis. Therefore we make approximations whenever necessary to simplify the mathematical form.

We first consider how the simplest loop extrusion model, which involves just 3 parameters, may facilitate DSB synapsis. After that, we extend this simple model by adding the four individual mechanisms discussed in the main text that improve synapsis efficiency: LEF stabilization by boundary elements (BEs); the presence of a subpopulation of long-lived LEFs; LEF stabilization by the DSB ends; and targeted loading of LEFs at DSB ends. Finally, we derive an analytical expression that combines all four additional mechanisms.

We note that our theory does not consider the effect of passive diffusion. Since passive 3D diffusion likely contributes to synapsis in cells in a manner that is synergistic with loop extrusion, we note that our estimates of DSB synapsis efficiencies should be considered lower bounds.

1.1 The probability of synapsis mediated by loop-extruding factors

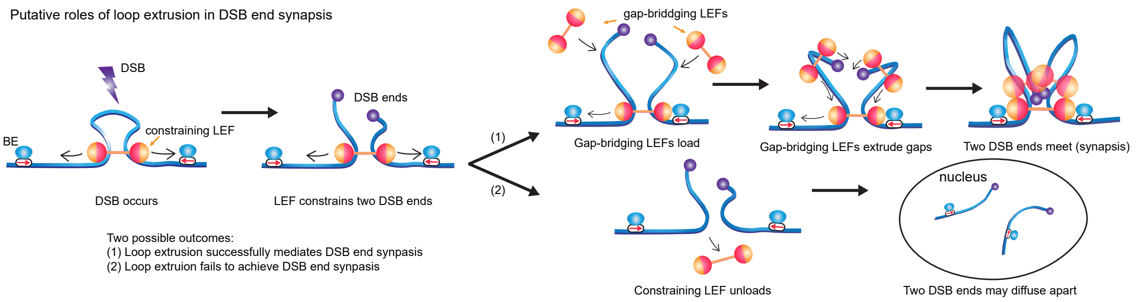

Pathway for successful LEF-mediated DSB synapsis. (1) Loop extrusion may facilitate successful DSB end synapsis in two steps: (i) the constraining LEF prevents the two DSB ends from diffusing apart after DSB and (ii) additional gap-bridging LEFs loaded within the loop extruded by constraining LEF can extrude sub-loops to bring the two DSB ends into proximity (2) If the constraining LEF falls off before the two DSB ends are brought into proximity by gap bridging LEFs, the two DSB ends may diffuse apart. In our simulations, we assume synapsis always fails once no constraining LEF remains for a given DSB.

We describe a general form for the probability of synapsis mediated by LEFs, P_{synapsis}. For the purpose of understanding the limitations of what LEFs can and cannot do for synapsis, we omit contributions from 3D diffusion in all subsequent calculations of P_{synapsis}.

Therefore, in order for LEF-mediated synapsis to happen, at least one constraining LEF must reside over the DSB site at the time when the DSB occurs. We denote the probability of having at least one constraining LEF as $P_{\text{constrained}}$, which can be calculated as the probability that the DSB occurs inside a DNA loop extruded by a LEF. If the condition of having at least one constraining LEF over the DSB is met, then additional LEFs loaded into the gap between the DSB and the edges of the constraining LEF can extrude loops to bring the DSB ends into proximity to achieve synapsis (path (1) in the figure above). We refer to this process as gap-bridging and the LEFs that mediate gap-bridging as gap-bridging LEFs. We note that at least one constraining LEF needs to remain throughout the gap-bridging process until synapsis is simultaneously achieved on both sides. If the constraining LEF dissociates before gap-bridging on both sides is achieved, we assume that the two DSBs ends can diffuse apart, and we consider this a failed LEF-mediated synapsis event (path (2) in the figure above). We denote the conditional probability of finishing gap-bridging given that the DSB ends are constrained at the time of DSB occurrence as $P_{\text{end-joining|constrained}}$.

Thus, the probability of synapsis, P_{synapsis}, can then be expressed as the product of $P_{\text{constrained}}$ and $P_{\text{end-joining|constrained}}$:

$$\boxed{P_{\text{synapsis}} = P_{\text{constrained}} \cdot P_{\text{end-joining|constrained}}}. \tag{1}$$

In the following sections, we derive analytical estimates for both $P_{\text{constrained}}$ and $P_{\text{end-joining|constrained}}$.

1.2 The probability of DSB ends being constrained

As stated above, $P_{\text{constrained}}$ is simply the probability that the DSB occurs inside a DNA loop such that the broken DSB ends remain constrained by at least one LEF. Since we assume that DSBs occur homogeneously throughout the genome, $P_{\text{constrained}}$ is equivalent to the fraction of the genome that is extruded into loops by LEFs. For the scenario without BEs, the fraction of the genome inside loops can be estimated by considering the nesting of LEFs [1]:

$$P_{\text{constrained,noBEs}} = 1 - e^{-\frac{l}{d}} \tag{2}$$

where l is the average DNA loop size, and d is the LEF separation, i.e., the average linear distance between LEF loading sites. This expression accounts for the increasing chance of LEFs loaded into existing loops and thus not contributing to increasing the fraction of genome inside loops.

To incorporate the effect of BEs on $P_{constrained}$, we account for how BEs decrease the amount of DNA that is inside loops by prematurely stalling LEFs. Therefore, the fraction of genome extruded into loops becomes:

$$P_{\text{constrained}} = P_{\text{constrained,noBEs}} \cdot (1 - P_{\text{unextruded,BEstalling}}) \tag{3}$$

where $P_{\text{unextruded,BEstalling}}$ is the probability of DNA becoming unextruded (i.e. unlooped) due to stalling of LEFs at BEs.

To calculate $P_{\text{unextruded,BEstalling}}$, we adapted the mean-field theoretical model previously derived by Banigan and Mirny [2] for the fraction of genome coverage by loops. Therefore,

$$\frac{(1 - P_{\text{unextruded,BEstalling}}) \cdot G_{\text{loop,noBEs}}}{N_p} = l \tag{4}$$

$$N_p l + N_p \cdot \sum_{\{i\}} f_{\text{unextruded},i} \cdot l_{\text{unextruded},i} = G_{\text{loop,noBEs}} \tag{5}$$

where $G_{\text{loop,noBEs}}$ is the average length of genome inside loops with no BEs present, and thus if the total genome length is G, $G_{\text{loop,noBEs}} = G \cdot P_{\text{constrained,noBEs}}$. The fraction of $G_{\text{loop,noBEs}}$ that remains looped in the presence of BEs is given by $1 - P_{\text{unextruded,BEstalling}}$. N_p is the total number of parent loops as defined in [2] (i.e. it is the total number of loops minus the number that exist within a larger loop). $f_{\text{unextruded},i}$ is the probability of adjacent parent LEFs being in a state i, where the set of possible $\{i\}$ constitutes all configurations of two adjacent LEFs and BEs which result in unextruded DNA between the LEFs because of the presence of BEs (see the figure below). Finally, $l_{\text{unextruded},i}$ is the average length of unextruded DNA for the configuration i. Here the size of parent loops are approximated as the average loop size of all LEFs. Combining Eqs.(4)-(5), we can solve for $P_{\text{unextruded,BEstalling}}$:

$$P_{\text{unextruded,BEstalling}} = 1 - \frac{1}{1 + \frac{\sum_{\{i\}} f_{\text{unextruded},i} \cdot l_{\text{unextruded},i}}{l}} \tag{6}$$

The unknown quantities now become $f_{\text{unextruded},i}$ and $l_{\text{unextruded},i}$, which we can calculate by enumerating the types of configurations which lead unextruded DNA because of a BE. Instead of explicitly enumerating and calculating all the possible configurations, we take a mean field approach and consider the leading three configurations as being representative of the much larger space of configurations.

We first consider the case where the loading sites of two adjacent LEFs are separated by a BE (i.e. are in adjacent topologically associated domains, (TADs)). We refer to this case as Configuration I, as shown in the figure below.

In our mean field approach, this scenario arises if the average distance between the BE and the loading site of LEFs (i.e. $\frac{d}{2}$) is smaller than the TAD size, D, i.e. $\frac{d}{2} \leq D$. In this case, unextruded DNA arises if and only if one LEF is stalled by BEs and the other LEF fails to reach BE. The probability of a LEF motor subunit being stalled by BEs can be estimated by the fraction, $\frac{[\text{BE-LEF}]}{[\text{LEF}_\text{o}]}$, where $[\text{BE-LEF}]$ is the concentration of stalled LEF-BE complexes and $[\text{LEF}_\text{o}]$ is the total density of LEF motor subunits extruding in one direction:

$$[\text{LEF}_\text{o}] = \frac{1}{d} \tag{7}$$

Thus the overall fraction of LEFs in *Configuration I* can be calculated as the following:

$$f_{\text{unextruded,Configuration I}} = 2\frac{[\text{BE-LEF}]}{[\text{LEF}_\text{o}]} P(M < \frac{d}{2}) \tag{8}$$

where M is the random variable representing the extrusion distance of one LEF motor subunit before the LEF unloads. The pre-factor of 2 accounts for the fact that the LEF stalled by BE could be either on the left or on the right of the BE. If we assume M is exponentially distributed, since each LEF motor subunit (a LEF is composed of

two subunits) on average travels $l/2$, then $M \sim \text{Exp}(l/2)$. Thus the probability that M is smaller than $\frac{d}{2}$ can be calculated as:

$$P(M < \frac{d}{2}) = \int_0^{d/2} \frac{2}{l} \cdot \exp(-\frac{2}{l} \cdot m) dm$$
$$= 1 - \exp(-\frac{d}{l}). \tag{9}$$

The corresponding average unextruded DNA length can be calculated as the following (panel (**a**) of the figure below):

$$l_{\text{unextruded,Configuration I}} = \frac{d}{2} - \int_0^{d/2} \frac{2}{l} m \cdot \exp(-\frac{2}{l} \cdot m) dm$$
$$= \frac{d}{2} - \frac{1}{2}(l - (d+l)\exp(-\frac{d}{l})). \tag{10}$$

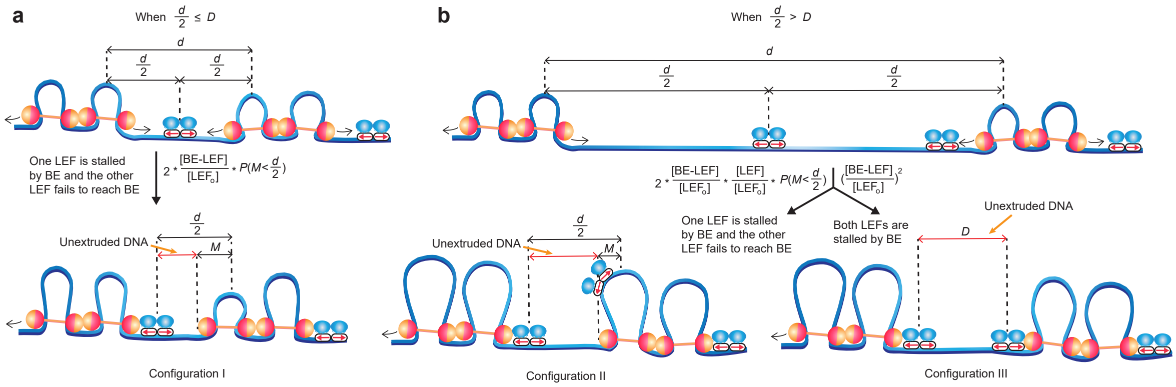

BE stalling can generate three types of LEF pair configurations leading to unextruded DNA. (**a**) When $\frac{d}{2} \leq D$, the loading sites of the LEF pair are in adjacent TADs, separated by a distance of d. On average, the BE is equidistant to both LEF loading sites. Unextruded DNA arises if and only and if one LEF is stalled by BE and the other LEF fails to reach BE, leading to an unextruded segment of average length calculated in Eq.(10).(**b**) When $\frac{d}{2} > D$, the loading sites of the LEF pair are separate by a TAD. Unextruded DNA can arise with two different LEF pair configurations. The first configuration is identical to the configuration in (A), which occurs when one LEF is stalled by BE, and the other LEF is not stalled by the additional BE in between but fails to reach the leftmost BE. The second configuration occurs when both LEFs are stalled by BEs, resulting in an unextruded segment with the same length as the TAD size.

Next we consider the cases where the loading sites of the adjacent LEFs are separated by a TAD (i.e. two BEs). This occurs when $\frac{d}{2} > D$. There are altogether two such types of configurations which we call Configuration II and Configuration III. Configuration II, occurs when one LEF is stalled by one of the BEs forming a BE-LEF complex, whereas the other LEF is not stalled, but fails to reach the BE-LEF complex thereby leaving an unextruded gap (left branch in panel (**b**) of the figure above). Let $[\text{LEF}]$ be the concentration of extruding LEFs not bound to BE. Configuration II type of scenarios occur with the following frequencies and result in the following lengths of unextruded DNA:

$$f_{\text{unextruded,Configuration II}} = 2\frac{[\text{BE-LEF}]}{[\text{LEF}_\text{o}]}\frac{[\text{LEF}]}{[\text{LEF}_\text{o}]}P(M < \frac{d}{2}) \tag{11}$$
$$= 2\frac{[\text{BE-LEF}]}{[\text{LEF}_\text{o}]}\frac{[\text{LEF}]}{[\text{LEF}_\text{o}]}(1 - \exp(-\frac{d}{l})) \tag{12}$$
$$l_{\text{unextruded,Configuration II}} = \frac{d}{2} - \int_0^{d/2} \frac{2}{l} m \cdot \exp(-\frac{2}{l} \cdot m) dm$$
$$= \frac{d}{2} - \frac{1}{2}(l - (d+l)\exp(-\frac{d}{l})). \tag{13}$$

Configuration III arises when both LEFs are each stalled by one of the BEs, leading to an unextruded DNA segment of average length that equals the TAD size D (right branch in panel (**b**) of the figure above). Note that while in reality there could be more than one TAD is in between the loading sites of two adjacent LEFs, we do not consider those scenarios within our parameter space under mean-field theoretical model, since the largest separation d we consider is 500 kb, and the smallest total size of two adjacent TADs in our simulations is 600 kb (200 kb and 400 kb

TAD next to each other), and thus on average there would not be more than one TAD between the loading sites of two adjacent LEFs. Configuration III type of scenarios occur with the following frequencies and result in the following lengths of unextruded DNA:

$$f_{\text{unextruded,Configuration III}} = (\frac{[\text{BE-LEF}]}{[\text{LEF}_\text{o}]})^2 \tag{14}$$

$$l_{\text{unextruded,Configuration III}} = D. \tag{15}$$

Combining Eqs.(2)-(3),Eqs.(6)-(15), we obtain the expression for $P_{\text{constrained}}$:

$$P_{\text{constrained}} = \begin{cases} (1-e^{-\frac{l}{d}})/(1+\frac{[\text{BE-LEF}]}{[\text{LEF}_\text{o}]}(1-e^{-\frac{d}{l}})(\frac{d}{l}-1+(\frac{d}{l}+1)e^{-\frac{d}{l}})) & \text{if } \frac{d}{2} \leq D \\ (1-e^{-\frac{l}{d}})/(1+(\frac{[\text{BE-LEF}]}{[\text{LEF}_\text{o}]}\frac{[\text{LEF}]}{[\text{LEF}_\text{o}]}(1-e^{-\frac{d}{l}})(\frac{d}{l}-1+(\frac{d}{l}+1)e^{-\frac{d}{l}})+(\frac{[\text{BE-LEF}]}{[\text{LEF}_\text{o}]})^2\frac{D}{l})) & \text{if } \frac{d}{2} > D \end{cases} \tag{16}$$

The average DNA loop size l has been previously estimated using processivity λ and separation d with $\sim 1\%$ precision for $\frac{\lambda}{d}\epsilon[10^{-1.5}, 10^{5.5}]$, by applying the following expression obtained through fitting a 7-th degree polynomial to simulation results [1]:

$$l \approx 10^a \cdot d \tag{17}$$

where:

$$a = -0.08238 + 0.7258z - 0.2514z^2 - 0.003995z^3 + 0.03445z^4 - 0.01077z^5 + 0.001371z^6 - 6.472 \cdot 10^{-5}z^7 \tag{18}$$

$$z = \log_{10}(\frac{\lambda}{d}) \tag{19}$$

Now the only unknown in Eq.(16) is $\frac{[\text{BE-LEF}]}{[\text{LEF}_\text{o}]}$. To calculate $\frac{[\text{BE-LEF}]}{[\text{LEF}_\text{o}]}$, we consider the following system:

$$\text{BE} + \text{LEF} \overset{k_{\text{association}}}{\rightarrow} \text{BE-LEF} \overset{k_{\text{dissociation}}}{\rightarrow} \text{BE} + \text{LEF} \tag{20}$$

where BE-LEF is the complex of BE and LEF formed upon a LEF being stalled by a BE, with rate constant $k_{\text{association}}$. LEF in complex with BE dissociates from the DNA and reloads on the genome with rate constant $k_{\text{dissociation}}$. We assume one BE can only associate with one LEF at a time. Although we consider two-sided LEFs, the extrusion along both directions are identical and independent processes, thus we can consider the model in Eq.(20) only for extrusion in one direction, without loss of generality.

Since the rate of BE-LEF formation is determined by the rate of collision between BE and LEF, the association rate constant $k_{\text{association}}$ can be written as the following:

$$k_{\text{association}} = v \cdot b \tag{21}$$

where v is the unobstructed extrusion speed in one direction (i.e., 1/2 the total extrusion speed), and b is boundary strength, defined as the probability of BE stalling LEF extrusion upon encountering (with probability $1-b$, LEF extrudes past BE).

Once a LEF is stalled by a BE, the average duration before the LEF dissociates from the DNA is given by $\frac{\lambda}{2v}$, where λ is the processivity, i.e., the average length of DNA extruded by an unobstructed LEF. Thus the dissociation rate constant $k_{\text{dissociation}}$ can be written as the following:

$$k_{\text{dissociation}} = \frac{2v}{\lambda} \tag{22}$$

We can write the following differential equation to describe the rate of formation of BE-LEF:

$$\frac{d[\text{BE-LEF}]}{dt} = k_{\text{association}} \cdot [\text{BE}] \cdot [\text{LEF}] - k_{\text{dissociation}} \cdot [\text{BE-LEF}] \tag{23}$$

At steady state, we have:

$$\frac{d[\text{BE-LEF}]}{dt} = 0 \tag{24}$$

Combining Eq.(21)-Eq.(24), we get:

$$\frac{[\text{BE-LEF}]}{[\text{LEF}_\text{o}]} = \begin{cases} \frac{d}{2D} + \frac{1}{2} + \frac{d}{b\lambda} - \sqrt{(\frac{d}{2D} + \frac{1}{2} + \frac{d}{b\lambda})^2 - \frac{d}{D}} & \text{if } b > 0 \\ 0 & \text{if } b = 0 \end{cases} \tag{25}$$

$$\frac{[\mathrm{LEF}]}{[\mathrm{LEF_o}]} = 1 - \frac{[\mathrm{BE\text{-}LEF}]}{[\mathrm{LEF_o}]} \tag{26}$$

Now we can substitute Eqs.(25)-(26) into Eq.(16) to solve for $P_{\mathrm{constrained}}$. In the simulations we have four different TAD sizes of $D_j \epsilon \{200\ \mathrm{kb}, 400\ \mathrm{kb}, 800\ \mathrm{kb}, 1200\ \mathrm{kb}\}$, each of which appears with a frequency of $\omega_j \epsilon \{0.5, 0.25, 0.125, 0.125\}$ respectively, and thus $P_{\mathrm{constrained}}$ can be computed by summing Eq.(16) with different TAD sizes weighted by the frequency of the TAD size (assuming $b > 0$):

$$P_{\mathrm{constrained}} = \sum_{j=1}^{4} \begin{cases} \omega_j (1 - e^{-\frac{l}{d}})/(1 + (\frac{d}{2D_j} + \frac{1}{2} + \frac{d}{b\lambda} - \sqrt{(\frac{d}{2D_j} + \frac{1}{2} + \frac{d}{b\lambda})^2 - \frac{d}{D_j}})(1 - e^{-\frac{d}{l}}) & \\ (\frac{d}{l} - 1 + (\frac{d}{l} + 1)e^{-\frac{d}{l}})) & \text{if } \frac{d}{2} \leq D_j \\ \omega_j (1 - e^{-\frac{l}{d}})/(1 + (\frac{d}{2D_j} + \frac{1}{2} + \frac{d}{b\lambda} - \sqrt{(\frac{d}{2D_j} + \frac{1}{2} + \frac{d}{b\lambda})^2 - \frac{d}{D_j}}) & \\ (\frac{1}{2} - \frac{d}{2D_j} - \frac{d}{b\lambda} + \sqrt{(\frac{d}{2D_j} + \frac{1}{2} + \frac{d}{b\lambda})^2 - \frac{d}{D_j}})(1 - e^{-\frac{d}{l}}) & \\ (\frac{d}{l} - 1 + (\frac{d}{l} + 1)e^{-\frac{d}{l}}) + (\frac{d}{2D_j} + \frac{1}{2} + \frac{d}{b\lambda} - \sqrt{(\frac{d}{2D_j} + \frac{1}{2} + \frac{d}{b\lambda})^2 - \frac{d}{D_j}})^2 \frac{D_j}{l})) & \text{if } \frac{d}{2} > D_j \end{cases} \tag{27}$$

The theoretical expression we derived for $P_{\mathrm{constrained}}$ in Eq.(27) can predict the percentage of DSB sites constrained by LEFs with reasonably high accuracy, as shown in the figure below. For the rest of the paper, we use the weighted average TAD size of $\bar{D} = \sum_{j=1}^{4} D_j \cdot \omega_j = 450\ \mathrm{kb}$ to simplify equations and calculations, unless specified otherwise.

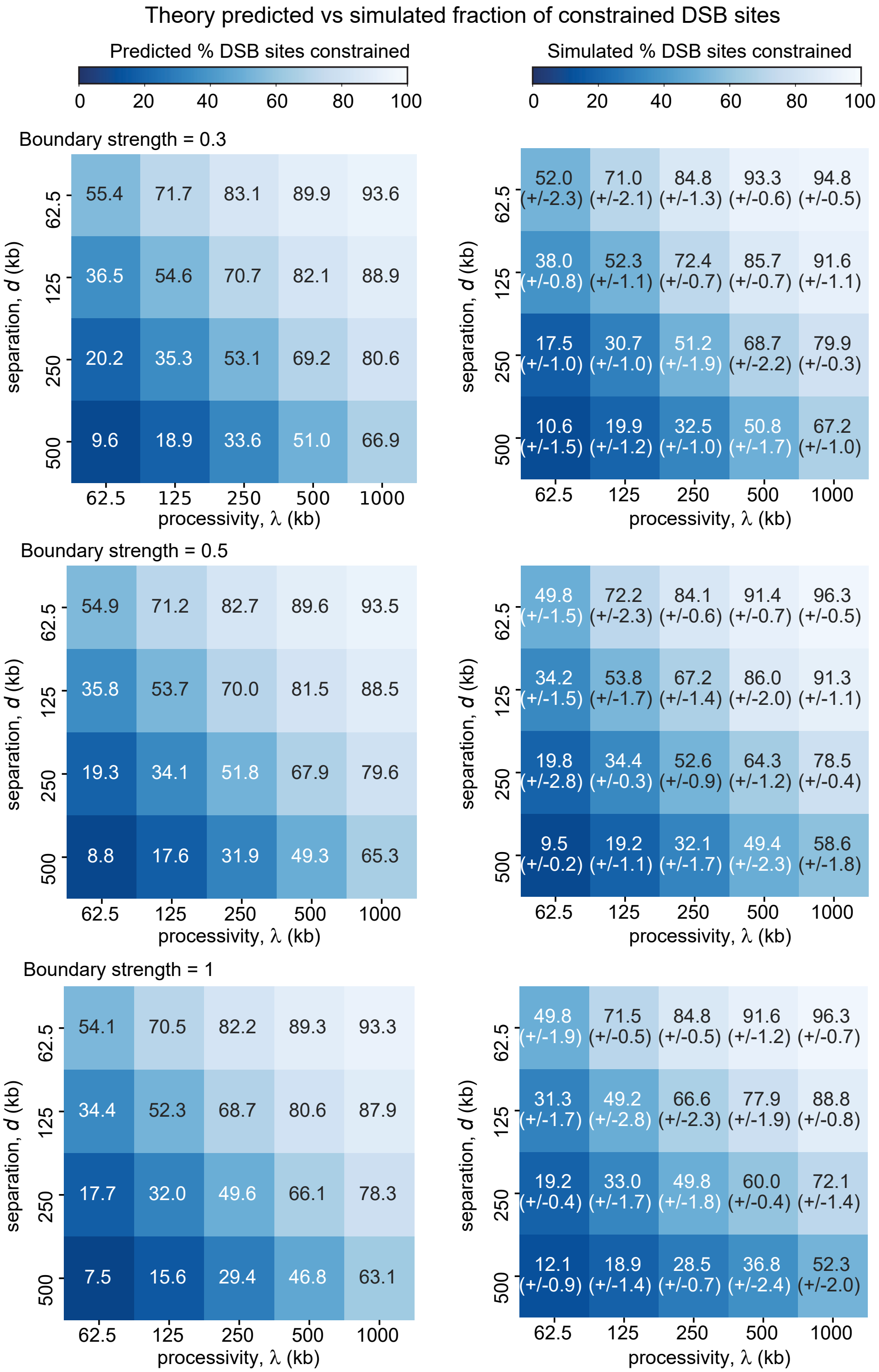

Theory prediction of the fraction of DSB sites constrained by LEFs is consistent with simulation results across different boundary strengths. The middle panel with boundary strength = 0.5 is the same as main text **Fig. 3b**. $P_{\mathrm{constrained}}$ can be predicted given processivity, separation, and boundary strength. Heatmaps of predicted (left) and simulated (right; numbers in brackets show standard error of mean, n=3 independent 1D simulations, with 218 DSB events per simulation) fraction of constrained DSB sites with different combinations of processivity (y axis) and separation (x axis). Each row corresponds to boundary strengths of 0.3, 0.5 and 1 respectively.

1.2.1 Simplified expression for the probability of DSB ends being constrained

By using a linear approximation (tangent line approximation) of $\frac{[\text{BE-LEF}]}{[\text{LEF}_\text{o}]}$, we can simplify the expression for $P_{\text{constrained}}$. To this end, we define:

$$h(x) = \sqrt{x} \tag{28}$$

Then the linear approximation of $h(x)$, $\mathrm{H}(x)$, can be written as:

$$\begin{aligned}\mathrm{H}(x) &= h(x_o) + h'(x_o)(x - x_o) \\ &= \sqrt{x_o} + \frac{1}{2\sqrt{x_o}}(x - x_o)\end{aligned} \tag{29}$$

Substituting $x = (\frac{d}{2\bar{D}} + \frac{1}{2} + \frac{d}{b\lambda})^2 - \frac{d}{\bar{D}}$ and $a = (\frac{d}{2\bar{D}} + \frac{1}{2} + \frac{d}{b\lambda})^2$ into Eq.(29), we obtain the linear approximation of Eq.(25):

$$\frac{[\text{BE-LEF}]}{[\text{LEF}_\text{o}]} \approx \begin{cases} \frac{1}{1+\frac{\bar{D}}{d}+\frac{2\bar{D}}{b\lambda}} & \text{if } b > 0 \\ 0 & \text{if } b = 0 \end{cases} \tag{30}$$

Substituting Eq.(26) and Eq.(30) into Eq.(16), we get the simplified expression for $P_{\text{constrained}}$:

$$P_{\text{constrained}} \approx \begin{cases} (1 - e^{-\frac{l}{d}})/(1 + \frac{(1-e^{-\frac{d}{l}})(\frac{d}{l}-1+(\frac{d}{l}+1)e^{-\frac{d}{l}})}{1+\frac{\bar{D}}{d}+\frac{2\bar{D}}{b\lambda}}) & \text{if } \frac{d}{2} \leq \bar{D} \\ (1 - e^{-\frac{l}{d}})/(1 + (\frac{(1-e^{-\frac{d}{l}})(\frac{\bar{D}}{d}+\frac{2\bar{D}}{b\lambda})(\frac{d}{l}-1+(\frac{d}{l}+1)e^{-\frac{d}{l}})+\frac{\bar{D}}{l}}{(1+\frac{\bar{D}}{d}+\frac{2\bar{D}}{b\lambda})^2})) & \text{if } \frac{d}{2} > \bar{D} \end{cases} \tag{31}$$

Eq.(31) shows $P_{\text{constrained}}$ is a monotonic increasing function of l and λ, and a monotonic decreasing function of d.

1.3 The probability of end joining given that DSB ends are constrained

To calculate the probability of gap-bridging given that DSB ends are constrained, $P_{\text{end-joining}|\text{constrained}}$, we want to compute how often simultaneous gap-bridging on both sides of the DSB happens before the constraining LEF unloads. In other words, we want to determine how frequently the time it takes to achieve synapsis is shorter than the lifetime of the constraining LEF. Since gap-bridging on both sides of the DSB are independent of each other, it is more mathematically tractable to consider the probability of gap-bridging on each side, and then take into account that synapsis requires the gap-bridging on both sides of the DSB to happen at the same time. Therefore, we need to formulate the time to bridge the gap and the lifetime of constraining LEFs respectively, which we will discuss next.

1.3.1 The gap-bridging time distribution

We define the gap-bridging time, T, as the duration between DSB occurrence and the first time that the gap is bridged on one side of the DSB. Thus, let the first-passage time, T, be a random variable and the probability distribution of T be given by f_T. To simplify the theory, we initially assume there is no gap-bridging LEFs present between the constraining LEF and DSB ends at the time of DSB occurrence (we modify this assumption later on considering the full probability of gap-bridging), and we assume only one gap-bridging LEF carries out the bridging from beginning to end. Therefore, we can conceptualize gap-bridging on one side of the DSB for a given DSB site as a two-step process: first, a gap-bridging LEF must load between the DSB end and the constraining LEF; second, the gap-bridging LEF must extrude to bridge the gap between the DSB end and the constraining LEF. We define the loading time random variable as X, corresponding to the time it takes to finish the first step, and the extrusion time random variable as Y, corresponding to the time it takes to complete the second step. Thus,

$$T = X + Y \tag{32}$$

1.3.1.1 The loading time distribution Generally, we can assume that the process of loading gap-bridging LEFs into the gap between DSB end is Markovian, and thus exponential, with parameter, $k_{\text{load}} = \langle\tau_{\text{load}}\rangle^{-1}$, where k_{load} is the LEF loading rate, and $\langle\tau_{\text{load}}\rangle$ is the average loading time. We assume LEFs reload randomly (uniform loading probability across the genome) as soon as they unload.

Let, L, be the length of the gap, i.e., the length of DNA between the DSB end and the edge of the constraining LEF. We assume the LEF lifetime is exponentially distributed with an average lifetime of $\frac{\lambda}{2v}$. The probability density function(PDF) of the loading time X, defined on the interval $x\epsilon[0, \infty)$, can be described by the following exponential function:

$$X \sim f_X(x) = k_{\text{load}} \exp(-k_{\text{load}} x) = \frac{1}{\langle\tau_{\text{load}}\rangle} \exp\left(-\frac{x}{\langle\tau_{\text{load}}\rangle}\right) \tag{33}$$

in which:

$$k_{\text{load}} = \frac{2v}{\lambda} \cdot \frac{L}{d} \tag{34}$$

where, as a reminder, v is the extrusion speed in one direction (i.e., 1/2 the total extrusion rate), λ is the processivity (i.e., the average length of DNA extruded by an unobstructed LEF), L is the length of the DNA between the DSB end and the edge of the constraining LEF, and d is the average linear distance between LEF loading sites.

As can be told from Eq.(34), the longer the gap, L, between the DSB and the edge of the constraining LEF, the faster the loading of a gap-bridging LEF.

1.3.1.2 The extrusion time distribution Provided that a gap-bridging LEF has been loaded in the gap, we can then determine the distribution of extrusion times. Since we assume that only one gap-bridging LEF extrudes the gap, the extrusion time is determined by the time it takes to extrude to whichever of the DSB and constraining LEF is furthest away: $\max(\frac{h}{v}, \frac{L-h}{v})$ where $h \epsilon [0, L]$ is the loading point of the LEF, as shown in the diagram below. Thus, the minimum extrusion time of $\frac{L}{2v}$, is achieved when the gap-bridging LEF loads right in the center of the gap, whereas the maximum extrusion time of $\frac{L}{v}$ is achieved when the gap-bridging LEF loads at either boundary of the gap. Since we assume the loading of the LEFs is spatially homogeneous, the extrusion time $Y \sim$ Uniform$(\frac{L}{2v}, \frac{L}{v})$.

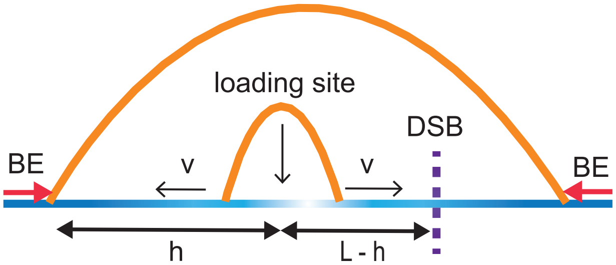

Thus, the PDF of the extrusion time Y is given by the following uniform distribution:

$$Y \sim f_Y(y) = \frac{1}{|\frac{L}{v} - \frac{L}{2v}|} = \frac{2v}{L} \tag{35}$$

defined on the interval $[\frac{L}{2v}, \frac{L}{v}]$.

We can rewrite Eq.(35) as the following to simplify expressions later on:

$$Y \sim f_Y(y) = \frac{2v}{L}\Theta\left(\frac{L}{2v} \leq y \leq \frac{L}{v}\right) \tag{36}$$

where,

$$\Theta = \begin{cases} 1 & \text{if the expression in the parentheses is True} \\ 0 & \text{otherwise} \end{cases} \tag{37}$$

1.3.1.3 Computing the gap-bridging time distribution through convolution Having determined the loading time distribution, f_X, and the extrusion time distribution, f_Y, we can now compute $T = X + Y$. To do so we need to compute the convolution of f_X and f_Y:

$$f_T(t) = \int_{-\infty}^{\infty} dx\, f_X(x) f_Y(t-x) \tag{38}$$

$$\begin{aligned} f_T(t) &= \int_{-\infty}^{\infty} dx\, f_X(x) f_Y(t-x) \\ &= \int_{-\infty}^{\infty} dx \left[\frac{2v}{L}\Theta\left(\frac{L}{2v} \leq t - x \leq \frac{L}{v}\right)\right] \left[k_{\text{load}} \exp\left(-k_{\text{load}} x\right) \Theta\left(x > 0\right)\right] \\ &= \frac{2v}{L}\int_{-\infty}^{\infty} dx \left[\Theta\left(t - \frac{L}{v} \leq x \leq t - \frac{L}{2v}\right)\right] \left[k_{\text{load}} \exp\left(-k_{\text{load}} x\right) \Theta\left(x > 0\right)\right] \\ &= \frac{2v}{L}\int_{\max(0, t-\frac{L}{v})}^{t-\frac{L}{2v}} dx\, k_{\text{load}} \exp\left(-k_{\text{load}} x\right) \end{aligned} \tag{39}$$

The solution has two parts, making it piece-wise continuous:

$$f_T(t) = \begin{cases} \frac{2v}{L}\int_0^{t-\frac{L}{2v}} dx\, k_{\text{load}} \exp\left(-k_{\text{load}} x\right) & \text{if t}<\frac{L}{v} \\ \frac{2v}{L}\int_{t-\frac{L}{v}}^{t-\frac{L}{2v}} dx\, k_{\text{load}} \exp\left(-k_{\text{load}} x\right) & \text{if t}\geq\frac{L}{v} \end{cases} \tag{40}$$

which results in:

$$f_T(t) = \begin{cases} \frac{2v}{L}(1 - \exp(-k_{\text{load}}(t - \frac{L}{2v})) & \text{if t}<\frac{L}{v} \\ \frac{2v}{L}(\exp(-k_{\text{load}}(t - \frac{L}{v})) - \exp(-k_{\text{load}}(t - \frac{L}{2v}))) & \text{if t}\geq\frac{L}{v} \end{cases} \tag{41}$$

1.3.2 The constraining LEF lifetime distribution

Since gap-bridging must take place before the constraining LEF unloads, we need to also compute the distribution of the constraining LEF lifetime. Let the lifetime of constraining LEFs be given by the random variable C. The probability distribution of C is given by f_C. As above, we assume the lifetime of constraining LEFs are exponentially distributed, and $\langle\tau_c\rangle$ is the average lifetime of constraining LEFs. The PDF of the constraining LEF lifetime C can be expressed as:

$$f_C(c) = \frac{1}{\langle\tau_c\rangle} \exp\left(-\frac{c}{\langle\tau_c\rangle}\right) \tag{42}$$

in which:

$$\langle\tau_c\rangle = \frac{\lambda}{2v} \tag{43}$$

1.3.3 The probability of gap-bridging on one side of the DSB

Having determined the gap-bridging time distribution f_T, and the constraining LEF lifetime distribution f_C, we can now compute the probability of gap-bridging on the side of the DSB that is bridged first. The calculation of f_T above assumes there is no gap-bridging LEF present at the time of DSB occurrence. However, there is a nonneglibible probability that gap-bridging LEFs are already present in the gap. The average number of gap-bridging LEFs within the constraining LEF, n, can be approximated as the following using Eq.(17) [1]:

$$n = \frac{l}{d} \approx 10^a \tag{44}$$

where a is defined in Eq.(18)-(19).

Let the number of gap-bridging LEFs inside the constraining LEF, N, be a random variable. Then N follows Poisson distribution:

$$N \sim \text{Pois}(n) \tag{45}$$

Thus the probability of having no gap-bridging LEFs inside the constraining LEF is:

$$\begin{aligned} P(N=0) &= \frac{n^0 e^{-\frac{l}{d}}}{0!} \\ &= e^{-n} \\ &= e^{-\frac{l}{d}} \end{aligned} \tag{46}$$

Conversely, the probability of having one or more gap-bridging LEFs is:

$$P(N \geq 1) = 1 - P(N=0) \tag{47}$$

$$= 1 - e^{-\frac{l}{d}} \tag{48}$$

As an approximation, we assume if one or more gap-bridging LEFs are present within the constraining LEF at the time of DSB occurrence, then one gap will be successfully bridged with probability 1. This approximation is motivated by the observation that the loading of gap-bridging LEF is the rate-limiting step of synapsis (**Supplementary Fig. 3**). If one or more gap-bridging LEFs are present within the constraining LEF, one of the two gaps are likely closed by the pre-existing gap-bridging LEFs. However, if initially there is no gap-bridging LEF inside the constraining LEF, the gap-bridging time T must be smaller than the constraining LEF lifetime C. We note f_T is a function of the gap length L, and we denote the gap length on the side bridged first as $L_{1^{st}}$. Therefore,

$$P_{1^{\text{st}}\text{gap-bridged}} = P(N \geq 1) \cdot 1 + P(N=0) \cdot \int_0^\infty dt\, f_T(T=t, L=L_{1^{st}}) P(C > t) \tag{49}$$

$$= 1 - e^{-\frac{l}{d}} + e^{-\frac{l}{d}} \int_0^\infty dt\, f_T(T=t, L=L_{1^{st}})(1 - F_C(t)) \tag{50}$$

where F_C is the cumulative distribution function of the constraining LEF lifetime:

$$F_C(t) = \int_{-\infty}^{t} dt' f_C(t'). \tag{51}$$

Because f_T is piece-wise continuous, we can compute this by breaking it up into two parts:

$$\begin{aligned}
P_{1^{\text{st}}\text{gap-bridged}} &= 1 - e^{-\frac{l}{d}} + e^{-\frac{l}{d}} \int_0^{\infty} dt\, f_T(t, L_{1^{st}}) \left[\int_t^{\infty} dt' f_C(t')\right] \\
&= 1 - e^{-\frac{l}{d}} + e^{-\frac{l}{d}} \cdot \\
&\left\{\int_0^{\frac{L_{1st}}{v}} dt\, f_T(t, L_{1^{st}}) \left[\int_t^{\frac{L_{1st}}{v}} dt' f_C(t')\right] + \int_{\frac{L_{1st}}{v}}^{\infty} dt\, f_T(t, L_{1^{st}}) \left[\int_t^{\infty} dt' f_C(t')\right]\right\} \\
&= 1 - e^{-\frac{l}{d}} + e^{-\frac{l}{d}} \frac{2v}{L_{1^{st}}} \cdot [\frac{\lambda}{2v}(e^{-\frac{L_{1st}}{\lambda}} - e^{-\frac{2L_{1st}}{\lambda}}) + \frac{e^{-\frac{2L_{1st}}{\lambda}} - e^{-\frac{L_{1st}}{\lambda}}}{k_{\text{load}} + \frac{2v}{\lambda}}]
\end{aligned} \tag{52}$$

1.3.4 Simultaneous gap-bridging on both sides of the DSB and the gap-bridging LEF lifetime distribution

In order to achieve synapsis, the gaps on both sides of the DSB must be bridged simultaneously. In other words, once the gap on one side of the DSB is bridged, the gap on the other side of the DSB must be bridged before the gap-bridging LEF on the side bridged first unloads. Let the lifetime of gap-bridging LEFs that have already finished gap-bridging on the side of DSB bridged first be given by the random variable G, whose PDF is f_G. Then the probability of gap bridging on the second side (while the gap-bridging LEF on the first side remains bound) can be written as:

$$P_{2^{\text{nd}}\text{gap-bridged}} = P(T \leq G \leq C) \tag{53}$$

$$= \int_0^{\infty} f_T(T = t, L = L_{2^{nd}}) P(C > t) P(G > t) dt \tag{54}$$

$$= \int_0^{\infty} dt f_T(T = t, L = L_{2^{nd}})(1 - F_C(t))(1 - F_G(t)) \tag{55}$$

where:

$$F_G(t) = \int_{-\infty}^{t} dt' f_G(t') \tag{56}$$

Utilizing the memoryless property of exponential distribution, the PDF of the lifetime G of the gap-bridging LEF that have already finished gap-bridging on the side bridged first, can be expressed as:

$$f_G(g) = \frac{1}{\langle \tau_g \rangle} \exp\left(-\frac{g}{\langle \tau_g \rangle}\right) \tag{57}$$

in which:

$$\langle \tau_g \rangle = \frac{\lambda}{2v} \tag{58}$$

Note the PDF of gap-bridging LEF lifetimes is mathematically identical to the PDF of constraining LEF lifetimes, since these two kinds of LEFs are essentially identical, and their identity are assigned based on their locations relative to a DSB. We assign different notations here to facilitate our discussions of extensions to the loop extrusion model later where distinguishing them becomes helpful. Now we can compute the probability of bridging the gap on the other side of the DSB while the gap-bridging LEF on the side bridged first remains:

$$\begin{aligned}
P_{2^{\text{nd}}\text{gap-bridged}} &= \int_0^{\infty} dt\, f_T(t, L_{2^{nd}}) \left[\int_t^{\infty} dt' f_C(t')\right] \left[\int_t^{\infty} dt' f_G(t')\right] \\
&= \frac{2v}{L_{2^{nd}}} \cdot [\frac{\lambda}{4v}(e^{-\frac{2L_{2nd}}{\lambda}} - e^{-\frac{4L_{2nd}}{\lambda}}) + \frac{e^{-\frac{4L_{2nd}}{\lambda}} - e^{-\frac{2L_{2nd}}{\lambda}}}{k_{\text{load}} + \frac{4v}{\lambda}}]
\end{aligned} \tag{59}$$

1.3.5 Computing the probability of joining DSB ends given DSB ends are constrained

Since gap-bridging on the first side and the second side of the DSB are independent processes, the probability of both gaps being bridged upon the first try is the product of Eq.(52) and Eq.(59). However, gap-bridging does not need to be achieved upon the first try: as long as the constraining LEF remains, even if the gap-bridging LEF on the side bridged first unloads, the gap-bridging process can continue until simultaneous gap-bridging on both sides of the DSB is fulfilled. Thus the probability of joining DSB ends given DSB ends are constrained for a given DSB site whose gap lengths are $L_{1^{st}}$ and $L_{2^{nd}}$ can be written as the following infinite sum:

$$\begin{aligned} P_{\text{end-joining}|\text{constrained}} = {} & [1 - e^{-\frac{l}{d}} + e^{-\frac{l}{d}} \cdot \int_0^\infty dt\, f_T(t, L_{1^{st}})(1 - F_C(t))] \cdot \\ & [\int_0^\infty dt\, f_T(t, L_{2^{nd}})(1 - F_C(t))(1 - F_G(t)) + \\ & \int_0^\infty dt\, f_T(t, L_{2^{nd}})(1 - F_C(t))F_G(t) \cdot \\ & \int_0^\infty dt\, f_T(t, L_{1^{st}})(1 - F_C(t))(1 - F_G(t)) + \\ & \int_0^\infty dt\, f_T(t, L_{2^{nd}})(1 - F_C(t))F_G(t) \cdot \\ & \int_0^\infty dt\, f_T(t, L_{1^{st}})(1 - F_C(t))F_G(t) \cdot \\ & \int_0^\infty dt\, f_T(t, L_{2^{nd}})(1 - F_C(t))(1 - F_G(t)) + ...] \end{aligned} \tag{60}$$

Utilizing the formula for the infinite sum of a geometric series, the equation above can be reduced to:

$$\begin{aligned} P_{\text{end-joining}|\text{constrained}} = {} & [1 - e^{-\frac{l}{d}} + e^{-\frac{l}{d}} \cdot \int_0^\infty dt\, f_T(t, L_{1^{st}})(1 - F_C(t))] \cdot \\ & \{\int_0^\infty dt\, f_T(t, L_{2^{nd}})(1 - F_C(t))(1 - F_G(t)) / \\ & [1 - \int_0^\infty dt\, f_T(t, L_{2^{nd}})(1 - F_C(t))F_G(t) \cdot \\ & \int_0^\infty dt\, f_T(t, L_{2^{nd}})(1 - F_C(t))F_G(t)] + \\ & \int_0^\infty dt\, f_T(t, L_{2^{nd}})(1 - F_C(t))(1 - F_G(t)) \cdot \\ & \int_0^\infty dt\, f_T(t, L_{2^{nd}})(1 - F_C(t))F_G(t) / \\ & [1 - \int_0^\infty dt\, f_T(t, L_{2^{nd}})(1 - F_C(t))F_G(t) \cdot \\ & \int_0^\infty dt\, f_T(t, L_{2^{nd}})(1 - F_C(t))F_G(t)]\} \end{aligned} \tag{61}$$

Eq.(61) is written for a specific DSB site with gap lengths of $L_{1^{st}}$ and $L_{2^{nd}}$ for the side bridged first and the side bridged second respectively. We assume the total length of the gaps on both sides of the DSB (the sum of $L_{1^{st}}$ and $L_{2^{nd}}$), is the average DNA loop size l:

$$L_{1^{st}} + L_{2^{nd}} = l \tag{62}$$

We can then integrate over $L_{1^{st}} \epsilon (0, l)$ to generalize Eq.(61) for any DSB site, given that the location of DSB is random:

$$P_{\text{end-joining}|\text{constrained}} = \frac{1}{l}\int_0^l dL_{1^{st}}\{1 - e^{-\frac{l}{d}} + e^{-\frac{l}{d}}\int_0^\infty dt\, f_T(T=t, L=L_{1^{st}})(1-F_C(t))\}\cdot$$
$$\{\int_0^\infty dt\, f_T(T=t, L=l-L_{1^{st}})(1-F_C(t))(1-F_G(t))/$$
$$[1 - \int_0^\infty dt\, f_T(T=t, L=l-L_{1^{st}})(1-F_C(t))F_G(t)\cdot$$
$$\int_0^\infty dt\, f_T(T=t, L=L_{1^{st}})(1-F_C(t))F_G(t)]+$$
$$\int_0^\infty dt\, f_T(T=t, L=L_{1^{st}})(1-F_C(t))(1-F_G(t))\cdot$$
$$\int_0^\infty dt\, f_T(T=t, L=l-L_{1^{st}})(1-F_C(t))F_G(t)/$$
$$[1 - \int_0^\infty dt\, f_T(T=t, L=l-L_{1^{st}})(1-F_C(t))F_G(t)\cdot$$
$$\int_0^\infty dt\, f_T(T=t, L=L_{1^{st}})(1-F_C(t))F_G(t)]\} \quad (63)$$

We define:

$$A(L) = \int_0^\infty f_T(T=t, L)(1-F_C(t))dt \quad (64)$$

$$B_1(L) = \int_0^\infty f_T(T=t, L)(1-F_C(t))(1-F_G(t))dt \quad (65)$$

$$B_2(L) = \int_0^\infty f_T(T=t, L)(1-F_C(t))F_G(t)dt \quad (66)$$

Thus Eq.(63) can be rewritten as:

$$P_{\text{end-joining}|\text{constrained}} = \frac{1}{l}\int_0^l [1 - e^{-\frac{l}{d}} + e^{-\frac{l}{d}}A(L_{1^{st}})]\cdot\{B_1(l-L_{1^{st}})/[1 - B_2(l-L_{1^{st}})\cdot B_2(L_{1^{st}})]+$$
$$B_1(L_{1^{st}})\cdot B_2(l-L_{1^{st}})/[1 - B_2(l-L_{1^{st}})\cdot B_2(L_{1^{st}})]\}dL_{1^{st}} \quad (67)$$

We have computed Eq.(64) and Eq.(65) for specific gap lengths of $L_{1^{st}}$ and $L_{2^{nd}}$ respectively in Eq.(52) and Eq.(59). We rewrite them below for the general gap length of L:

$$A(L) = \frac{2v}{L}(e^{-\frac{L}{\lambda}} - e^{-\frac{2L}{\lambda}})(\frac{\lambda}{2v} - \frac{1}{k_{\text{load}} + \frac{2v}{\lambda}}) \quad (68)$$

$$B_1(L) = \frac{2v}{L}(e^{-\frac{2L}{\lambda}} - e^{-\frac{4L}{\lambda}})(\frac{\lambda}{4v} - \frac{1}{k_{\text{load}} + \frac{4v}{\lambda}}) \quad (69)$$

Notice Eq.(66) is simply the difference between $A(L)$ and $B_1(L)$:

$$B_2(L) = A(L) - B_1(L) \quad (70)$$

Now we can substitute Eqs.(17)-(19) and Eqs.(68)-(70) into Eq.(67) and perform numerical integration to obtain $P_{\text{end-joining}|\text{constrained}}$.

The probability of synapsis P_{synapsis} can then be determined by multiplying the numerically integrated $P_{\text{end-joining}|\text{constrained}}$ and the $P_{\text{constrained}}$ computed in Eq.(27). Note that $P_{\text{end-joining}|\text{constrained}}$ and $P_{\text{constrained}}$ both only depend on the LEF processivity λ and the LEF separation d, and thus P_{synapsis} can also be determined as long as we know λ and d. Below we compare the synapsis efficiency predicted by our analytical expression for P_{synapsis} and the simulated synapsis efficiency with different combinations of λ and d. This comparison shows that our theory is reasonably accurate:

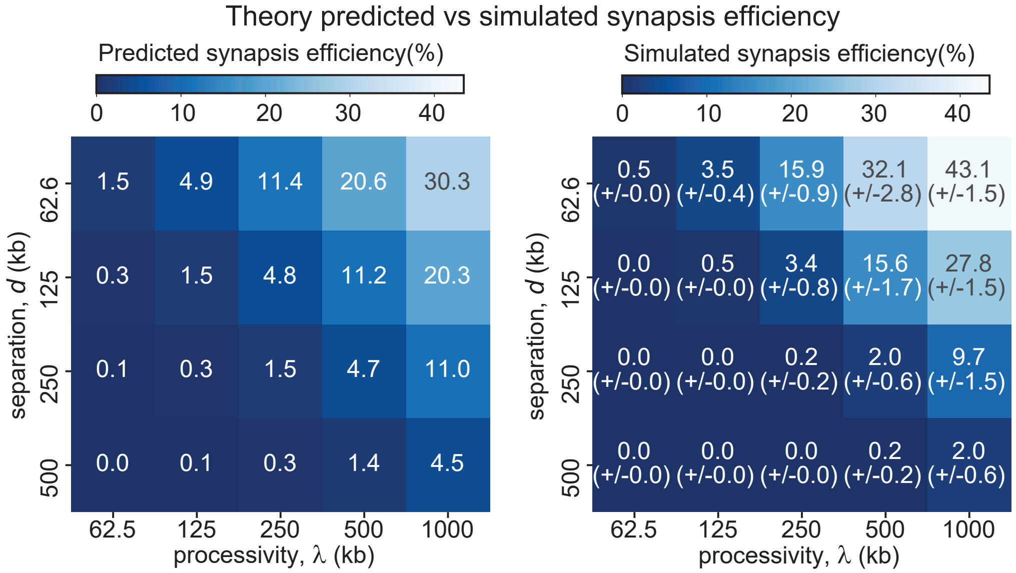

Theory prediction of synapsis efficiency is consistent with simulation results. Heatmaps of predicted (left) and simulated (right; numbers in brackets show standard error of mean, n=3 independent 1D simulations, with 218 DSB events per simulation) synapsis efficiency with different combinations of processivity (y axis) and separation (x axis). Boundary strength = 0.5 was used in the simulations.

1.3.6 Simplified expression for the probability of end-joining given the DSB is constrained and two important relative timescales

Our theory above highlights that P_{synapsis} can be determined from just λ and d. However, despite this seemingly simple picture and the reasonable agreement between our theory and simulations, our theoretical formulation for P_{synapsis} is still too complicated to be written in a compact form. Therefore, it is challenging for someone to gain mechanistic intuition for what factors underlie efficient synapsis. In this section, we aim to simplify the expression for P_{synapsis} with the goal of gaining mechanistic intuition.

Let us consider the scenario where gaps are bridged upon the first try. Further, let us consider a DSB site with the break right in the center of the constraining LEF, and thus with gap length $L = \frac{l}{2}$ on both sides of the DSB. We assume there is no gap-bridging LEF present in the constraining LEF at the time of DSB occurrence. Then $P_{\text{end-joining}|\text{constrained}}$ can be approximated as the following expression simplified from the product of Eq.(52) and Eq.(59):

$$\begin{aligned} P_{\text{end-joining}|\text{constrained}} &\approx A(\frac{l}{2}) \cdot B_1(\frac{l}{2}) \\ &= \frac{v}{l}(e^{-\frac{l}{2\lambda}} - e^{-\frac{l}{\lambda}})(\frac{\lambda}{2v} - \frac{1}{k_{\text{load}} + \frac{2v}{\lambda}}) \cdot \\ &\quad \frac{v}{l}(e^{-\frac{l}{\lambda}} - e^{-\frac{2l}{\lambda}})(\frac{\lambda}{4v} - \frac{1}{k_{\text{load}} + \frac{4v}{\lambda}}) \end{aligned} \tag{71}$$

We can rewrite Eq.(71) as:

$$\begin{aligned} P_{\text{end-joining}|\text{constrained}} \approx &[\frac{\tau_{\text{constrained}}^2}{\tau_{\text{extrusion}}(\tau_{\text{loading}} + \tau_{\text{constrained}})}(e^{-\frac{\tau_{\text{extrusion}}}{\tau_{\text{constrained}}}} - e^{-\frac{2\tau_{\text{extrusion}}}{\tau_{\text{constrained}}}})] \cdot \\ &[\frac{\tau_{\text{constrained}}^2}{2\tau_{\text{extrusion}}(2\tau_{\text{loading}} + \tau_{\text{constrained}})}(e^{-\frac{2\tau_{\text{extrusion}}}{\tau_{\text{constrained}}}} - e^{-\frac{4\tau_{\text{extrusion}}}{\tau_{\text{constrained}}}})] \end{aligned} \tag{72}$$

where:

$$\tau_{\text{constrained}} = \frac{\lambda}{2v} \tag{73}$$

$$\tau_{\text{extrusion}} = \frac{l}{4v} \tag{74}$$

$$\begin{aligned} \tau_{\text{loading}} &= \frac{1}{k_{\text{load}}} \\ &= \frac{\lambda d}{lv} \end{aligned} \tag{75}$$

We can further simplify Eq.(72) by defining:

$$f(a) = \frac{1}{a}(e^{-a} - e^{-2a}) \tag{76}$$

Then Eq.(72) can be written as:

$$P_{\text{end-joining}|\text{constrained}} \approx \frac{f(\frac{\tau_{\text{extrusion}}}{\tau_{\text{constrained}}})}{1 + \frac{\tau_{\text{loading}}}{\tau_{\text{constrained}}}} \cdot \frac{f(\frac{2\tau_{\text{extrusion}}}{\tau_{\text{constrained}}})}{1 + \frac{2\tau_{\text{loading}}}{\tau_{\text{constrained}}}}. \tag{77}$$

The simplified formula for $P_{\text{gap-bridged}|\text{constrained}}$ suggests that given the DSB ends are constrained, synapsis efficiency is dictated by two relative timescales: the ratio of loading time and the constraining LEF lifetime and the ratio of extrusion time and the constraining LEF lifetime. Further, $P_{\text{gap-bridged}|\text{constrained}}$ monotonically decreases with the two relative timescales, so decreasing either or both of the two relative timescales will improve synapsis efficiency.

Substituting Eq.(31) and Eq.(77) into Eq.(1), we can obtain a final simplified expression for the synapsis efficiency:

$$P_{\text{synapsis}} \approx \begin{cases} (1 - e^{-\frac{l}{d}})/(1 + \frac{(1-e^{-\frac{d}{l}})(\frac{d}{l}-1+(\frac{d}{l}+1)e^{-\frac{d}{l}})}{1+\frac{\bar{D}}{d}+\frac{2\bar{D}}{b\lambda}}) \cdot \frac{f(\frac{\tau_{\text{extrusion}}}{\tau_{\text{constrained}}})}{1+\frac{\tau_{\text{loading}}}{\tau_{\text{constrained}}}} \cdot \frac{f(\frac{2\tau_{\text{extrusion}}}{\tau_{\text{constrained}}})}{1+\frac{2\tau_{\text{loading}}}{\tau_{\text{constrained}}}} & \text{if } \frac{d}{2} \leq \bar{D} \\ (1 - e^{-\frac{l}{d}})/(1 + (\frac{(1-e^{-\frac{d}{l}})(\frac{\bar{D}}{d}+\frac{2\bar{D}}{b\lambda})(\frac{d}{l}-1+(\frac{d}{l}+1)e^{-\frac{d}{l}})+\frac{\bar{D}}{l}}{(1+\frac{\bar{D}}{d}+\frac{2\bar{D}}{b\lambda})^2})) \cdot \frac{f(\frac{\tau_{\text{extrusion}}}{\tau_{\text{constrained}}})}{1+\frac{\tau_{\text{loading}}}{\tau_{\text{constrained}}}} \cdot \frac{f(\frac{2\tau_{\text{extrusion}}}{\tau_{\text{constrained}}})}{1+\frac{2\tau_{\text{loading}}}{\tau_{\text{constrained}}}} & \text{if } \frac{d}{2} > \bar{D} \end{cases} \tag{78}$$

1.4 Synapsis with additional mechanisms

In this section, we will discuss four experimentally plausible extensions of the simple loop extrusion model that improve synapsis efficiency: stabilization of LEFs by BEs, the presence of a small fraction of long-lived LEFs, stabilization of LEFs by DSB ends, and targeted loading of LEFs to DSBs. We start by investigating how the addition of each of the four mechanisms modifies the expressions derived above. Finally, we end by showing the combined effect of all four mechanisms on synapsis efficiency.

1.4.1 Synapsis with stabilized LEFs by BEs

One mechanism that improves synapsis efficiency by increasing $P_{\text{constrained}}$ is the stabilization of LEFs by BEs. We define, w, as the fold increase in LEF lifetime through stabilization by BEs. We assume LEFs with one or both motors bound to a BE have identical fold increase in lifetime, w. Intuitively, $P_{\text{constrained}}$ increases with BE stabilization of LEFs since the LEFs stabilized by BEs extrude larger loops; therefore, DSBs are more likely to happen inside loops. To incorporate the effect of BE stabilization on $P_{\text{constrained}}$, we modify the rate of BE-LEF dissociation in Eq.(22) to the following:

$$k_{dissociation,\text{BEstabilized}} = \frac{2v}{w\lambda} \tag{79}$$

The updated BE-LEF dissociation rate in Eq.(79) in turn updates the fraction of LEF motor subunits extruding in one direction that are bound to BEs in Eqs.(25)-(26) to the following:

$$\frac{[\text{BE-LEF}]}{[\text{LEF}_\text{o}]}_{\text{BEstabilized}} = \begin{cases} \frac{d}{2\bar{D}} + \frac{1}{2} + \frac{d}{bw\lambda} - \sqrt{(\frac{d}{2\bar{D}} + \frac{1}{2} + \frac{d}{bw\lambda})^2 - \frac{d}{\bar{D}}} & \text{if } b > 0 \\ 0 & \text{if } b = 0 \end{cases} \tag{80}$$

$$\frac{[\text{LEF}]}{[\text{LEF}_\text{o}]}_{\text{BEstabilized}} = 1 - \frac{[\text{BE-LEF}]}{[\text{LEF}_\text{o}]}_{\text{BEstabilized}} \tag{81}$$

To account for the effect of BE stabilization of LEFs on parameters such as the average LEF processivity and average LEF loop size, we first need to know what fraction of LEFs are stalled at BEs. Let, β, be the fraction of LEFs that have at least one motor subunit bound to BEs, then β can be calculated as:

$$\beta = 1 - \left(1 - \frac{[\text{BE-LEF}]}{[\text{LEF}_\text{o}]}_{\text{BEstabilized}}\right)^2 \tag{82}$$

Eq.(82) can reasonably well predict the fraction of LEFs stabilized by BEs:

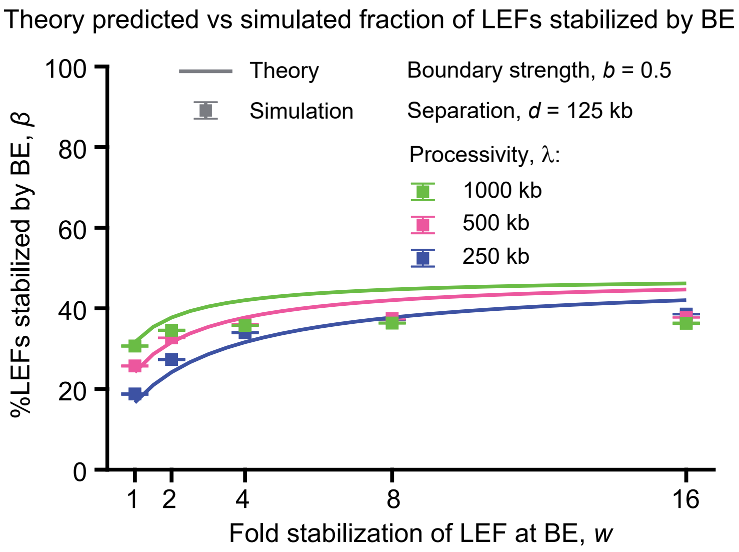

The percentage of LEFs stabilized by BEs predicted by our theory is largely consistent with the simulation results. The error bars represent the standard error of mean, n = 3 independent 1D simulations, with 218 DSB events per simulation.

We can then use Eq.(82) to compute the weighted average processivity and the calculate the average loop size:

$$\lambda_{\text{BEstabilized}} = \beta \cdot w\lambda + (1-\beta)\cdot\lambda \tag{83}$$

$$z_{\text{BEstabilized}} = \log_{10}\left(\frac{\lambda_{\text{BEstabilized}}}{d}\right) \tag{84}$$

$$\begin{aligned} a_{\text{BEstabilized}} &= -0.08238 + 0.7258 z_{\text{BEstabilized}} - 0.2514 z^2_{\text{BEstabilized}} \\ &\quad - 0.003995 z^3_{\text{BEstabilized}} + 0.03445 z^4_{\text{BEstabilized}} - 0.01077 z^5_{\text{BEstabilized}} \\ &\quad + 0.001371 z^6_{\text{BEstabilized}} - 6.472\cdot 10^{-5} z^7_{\text{BEstabilized}} \end{aligned} \tag{85}$$

$$l_{\text{BEstabilized}} = 10^{a_{\text{BEstabilized}}}\cdot d \tag{86}$$

Eqs.(80)-(81) and Eq.(86) in turn update the probability of DSB happening in loops defined in Eq.(16) to the following:

$$P_{\text{constrained,BEstabilized}} = \begin{cases} (1-e^{-\frac{l_{\text{BEstabilized}}}{d}})/(1+\frac{[\text{BE-LEF}]}{[\text{LEF}_\text{o}]}_{\text{BEstabilized}}(1-e^{-\frac{d}{l_{\text{BEstabilized}}}})\cdot \\ (\frac{d}{l_{\text{BEstabilized}}}-1+(\frac{d}{l_{\text{BEstabilized}}}+1)e^{-\frac{d}{l_{\text{BEstabilized}}}})) & \text{if } \frac{d}{2}\le\bar{D} \\ (1-e^{-\frac{l_{\text{BEstabilized}}}{d}})/(1+\frac{[\text{BE-LEF}]}{[\text{LEF}_\text{o}]}_{\text{BEstabilized}}\frac{[\text{LEF}]}{[\text{LEF}_\text{o}]}_{\text{BEstabilized}}(1-e^{-\frac{d}{l_{\text{BEstabilized}}}})\cdot \\ (\frac{d}{l_{\text{BEstabilized}}}-1+(\frac{d}{l_{\text{BEstabilized}}}+1)e^{-\frac{d}{l_{\text{BEstabilized}}}})+(\frac{[\text{BE-LEF}]}{[\text{LEF}_\text{o}]}_{\text{BEstabilized}})^2\frac{\bar{D}}{l_{\text{BEstabilized}}}) & \text{if } \frac{d}{2}>\bar{D} \end{cases} \tag{87}$$

We next aim to compute $P_{\text{end-joining}|\text{constrained,BEstabilized}}$. The first consideration is to account for the effect of BE stabilization on the distribution of constraining LEF lifetimes, f_C. We thus sought to calculate the probability that a DSB is flanked by constraining LEFs. As a first-pass, we calculate the probability that a DSB occurs in a TAD held together by at least one constraining LEF. Since a TAD is defined by a pair of convergent BEs, the probability,$P_{\text{stabilized}}$, of a TAD having at least one BE occupied, is:

$$P_{\text{stabilized}} = 1-\left(1-\frac{[\text{BE-LEF}]}{[\text{BE}_\text{o}]}_{\text{BEstabilized}}\right)^2 \tag{88}$$

Eq.(88) can accurately predict the fraction of TADs with stabilized LEFs:

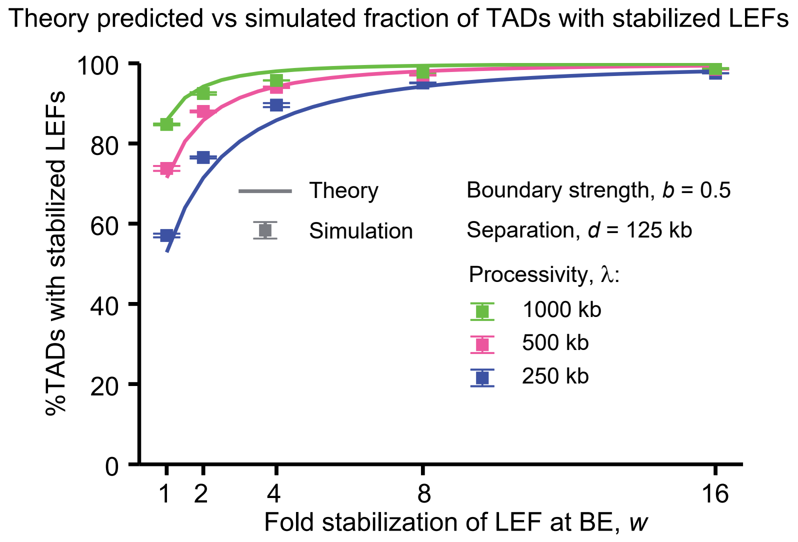

Most TADs have at least one LEF stabilized by BE. The error bars represent the standard error of mean, n = 3 independent 1D simulations, with 218 DSB events per simulation.

As shown in the figure above, most of TADs have at least one LEF stabilized by BE in the parameter space considered. Since the LEFs stabilized by BE will likely constrain DSB ends in a DSB-containing TAD due to their prolonged lifetime, we make the simplifying assumption that with LEF stabilization by BE, all constraining LEFs obtain a w fold increase in lifetime.

Thus, the modified constraining LEF lifetime distribution is:

$$f_{C,\text{BEstabilized}}(c) = \frac{1}{\langle \tau_{c,\text{BEstabilized}} \rangle} \exp\left(-\frac{c}{\langle \tau_{c,\text{BEstabilized}} \rangle}\right) \tag{89}$$

where:

$$\langle \tau_{c,\text{BEstabilized}} \rangle = \frac{w\lambda}{2v} \tag{90}$$

As a further simplifying approximation, we neglect the effect of BE stabilization on loading time distribution, since only the fraction of LEFs stabilized by BEs have slower dynamics.

The modified f_C updates Eqs.(67)-(70) to the following:

$$\begin{aligned} P_{\text{end-joining}|\text{constrained,BEstabilized}} = \frac{1}{l}\int_0^l & [1 - e^{-\frac{l}{d}} + e^{-\frac{l}{d}} A_{\text{BEstabilized}}(L_{1^{st}})] \cdot \{B_{1,\text{BEstabilized}}(l - L_{1^{st}}) / \\ & [1 - B_{2,\text{BEstabilized}}(l - L_{1^{st}}) \cdot B_{2,\text{BEstabilized}}(L_{1^{st}})] + B_{1,\text{BEstabilized}}(L_{1^{st}}) \cdot \\ & B_{2,\text{BEstabilized}}(l - L_{1^{st}}) / [1 - B_{2,\text{BEstabilized}}(l - L_{1^{st}}) \cdot B_{2,\text{BEstabilized}}(L_{1^{st}})]\} dL_{1^{st}} \end{aligned} \tag{91}$$

$$A_{\text{BEstabilized}}(L) = \frac{2v}{L}(e^{-\frac{L}{w\lambda}} - e^{-\frac{2L}{w\lambda}})(\frac{w\lambda}{2v} - \frac{1}{k_{\text{load}} + \frac{2v}{w\lambda}}) \tag{92}$$

$$B_{1,\text{BEstabilized}}(L) = \frac{2v}{L}(e^{-\frac{L}{\lambda}\cdot\frac{1+w}{w}} - e^{-\frac{2L}{\lambda}\cdot\frac{1+w}{w}})(\frac{w\lambda}{2v(1+w)} - \frac{1}{k_{\text{load}} + \frac{2v}{\lambda}\cdot\frac{1+w}{w}}) \tag{93}$$

$$B_{2,\text{BEstabilized}}(L) = A_{\text{BEstabilized}}(L) - B_{1,\text{BEstabilized}}(L) \tag{94}$$

$P_{\text{synapsis,BEstabilized}}$ can then be computed as:

$$P_{\text{synapsis,BEstabilized}} = P_{\text{end-joining}|\text{constrained,BEstabilized}} \cdot P_{\text{constrained,BEstabilized}} \tag{95}$$

Eq.(95) can accurately predict the synapsis efficiency with BE stabilization, validating our mechanistic explanation of BE stabilization facilitating synapsis by improving the chance of DSB happening inside a loop and increasing the lifetime of constraining LEFs:

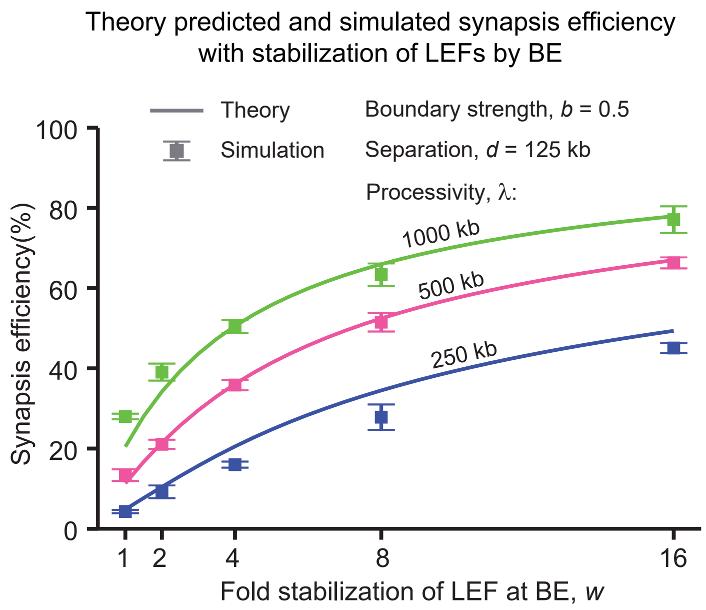

Theory predicts the synapsis efficiency with BE stabilization. Same as the bottom panel in main text **Fig. 4a**. The error bars represent the standard error of mean, n = 3 independent 1D simulations, with 218 DSB events per simulation.

1.4.2 Synapsis with a small fraction of long-lived LEFs

Another mechanism that increases both the chance of DSB happening in loops and $\tau_{\text{constrained}}$ is the presence of a small fraction of long-lived LEFs. Let, α_o, be the fraction of long-lived LEFs. Let, s, be the fold increase in long-lived LEFs' lifetime compared with normal LEFs. To incorporate the effect of the presence of a small fraction of long-lived LEFs on $P_{\text{constrained}}$, we use an weighted average processivity to update the average loop size and the fraction of LEF motors extruding in one direction that are bound to BEs:

$$\lambda_{\text{long-lived}} = \alpha_o \cdot s\lambda + (1-\alpha_o)\cdot\lambda \tag{96}$$

$$z_{\text{long-lived}} = \log_{10}\left(\frac{\lambda_{\text{long-lived}}}{d}\right) \tag{97}$$

$$\begin{aligned} a_{\text{long-lived}} = &-0.08238 + 0.7258 z_{\text{long-lived}} - 0.2514 z^2_{\text{long-lived}} \\ &- 0.003995 z^3_{\text{long-lived}} + 0.03445 z^4_{\text{long-lived}} - 0.01077 z^5_{\text{long-lived}} \\ &+ 0.001371 z^6_{\text{long-lived}} - 6.472\cdot 10^{-5} z^7_{\text{long-lived}} \end{aligned} \tag{98}$$

$$l_{\text{long-lived}} = 10^{a_{\text{long-lived}}}\cdot d \tag{99}$$

$$\frac{[\text{BE-LEF}]}{[\text{LEF}_\text{o}]}_{\text{long-lived}} = \begin{cases} \frac{d}{2D} + \frac{1}{2} + \frac{d}{b\lambda_{\text{long-lived}}} - \sqrt{\left(\frac{d}{2D} + \frac{1}{2} + \frac{d}{b\lambda_{\text{long-lived}}}\right)^2 - \frac{d}{D}} & \text{if } b>0 \\ 0 & \text{if } b=0 \end{cases} \tag{100}$$

$$\frac{[\text{LEF}]}{[\text{LEF}_\text{o}]}_{\text{long-lived}} = 1 - \frac{[\text{BE-LEF}]}{[\text{LEF}_\text{o}]}_{\text{long-lived}} \tag{101}$$

Eqs.(99)-(101) in turn update the probability of a DSB happening in loops defined in Eq.(16) to the following:

$$P_{\text{constrained,long-lived}} =$$

$$\begin{cases} (1-e^{-\frac{l_{\text{long-lived}}}{d}})/(1+\frac{[\text{BE-LEF}]}{[\text{LEF}_\text{o}]}_{\text{long-lived}}(1-e^{-\frac{d}{l_{\text{long-lived}}}})\cdot \\ \quad (\frac{d}{l_{\text{long-lived}}} - 1 + (\frac{d}{l_{\text{long-lived}}}+1)e^{-\frac{d}{l_{\text{long-lived}}}})) & \text{if } \frac{d}{2} \le \bar{D} \\ (1-e^{-\frac{l_{\text{long-lived}}}{d}})/(1+\frac{[\text{BE-LEF}]}{[\text{LEF}_\text{o}]}_{\text{long-lived}}\frac{[\text{LEF}]}{[\text{LEF}_\text{o}]}_{\text{long-lived}}(1-e^{-\frac{d}{l_{\text{long-lived}}}})\cdot \\ \quad (\frac{d}{l_{\text{long-lived}}} - 1 + (\frac{d}{l_{\text{long-lived}}}+1)e^{-\frac{d}{l_{\text{long-lived}}}}) + (\frac{[\text{BE-LEF}]}{[\text{LEF}_\text{o}]}_{\text{long-lived}})^2\frac{\bar{D}}{l_{\text{long-lived}}}) & \text{if } \frac{d}{2} > \bar{D} \end{cases} \tag{102}$$

Long-lived LEFs are over-represented in the population of constraining LEFs as they extrude larger loops. We therefore approximate the fraction of constraining LEFs that are long-lived LEFs, α, as the fraction of DNA extruded by long-lived LEFs among the DNA extruded by all LEFs assuming all LEFs are unobstructed:

$$\alpha \approx \frac{\alpha_o \cdot s}{(1-\alpha_o)+\alpha_o \cdot s} \tag{103}$$

Then PDF of the constraining lifetime will be modified to the following:

$$f_{C,\text{long-lived}}(c) = (1-\alpha)\cdot \frac{1}{\langle \tau_c \rangle} \exp\left(-\frac{c}{\langle \tau_c \rangle}\right) + \alpha \cdot \frac{1}{\langle \tau_{c,\text{long-lived}} \rangle} \exp\left(-\frac{d}{\langle \tau_{c,\text{long-lived}} \rangle}\right) \tag{104}$$

where:

$$\langle \tau_{c,\text{long-lived}} \rangle = \frac{s\lambda}{2v} \tag{105}$$

As we only consider a small fraction of long-lived LEFs, we neglect its effect on the loading time distribution as a simplifying approximation.

The modified f_C updates Eqs.(67)-(70) to the following:

$$\begin{aligned} P_{\text{end-joining}|\text{constrained,long-lived}} = \frac{1}{l}\int_0^l [1-e^{-\frac{l}{d}} + e^{-\frac{l}{d}} A_{\text{long-lived}}(L_{1^{st}})] \cdot \{B_{1,\text{long-lived}}(l-L_{1^{st}})/ \\ [1-B_{2,\text{long-lived}}(l-L_{1^{st}}) \cdot B_{2,\text{long-lived}}(L_{1^{st}})] + B_{1,\text{long-lived}}(L_{1^{st}}) \cdot \\ B_{2,\text{long-lived}}(l-L_{1^{st}})/[1-B_{2,\text{long-lived}}(l-L_{1^{st}}) \cdot B_{2,\text{long-lived}}(L_{1^{st}})]\} dL_{1^{st}} \end{aligned} \tag{106}$$

$$\begin{aligned} A_{\text{long-lived}}(L) = \frac{2v}{L}[\alpha(e^{-\frac{L}{s\lambda}} - e^{-\frac{2L}{s\lambda}})(\frac{s\lambda}{2v} - \frac{1}{k_{\text{load}} + \frac{2v}{s\lambda}})+ \\ (1-\alpha)(e^{-\frac{L}{\lambda}} - e^{-\frac{2L}{\lambda}})(\frac{\lambda}{2v} - \frac{1}{k_{\text{load}} + \frac{2v}{\lambda}})] \end{aligned} \tag{107}$$

$$\begin{aligned} B_{1,\text{long-lived}}(L) = \frac{2v}{L}[\alpha(e^{-\frac{L}{\lambda}\cdot\frac{s+1}{s}} - e^{-\frac{2L}{\lambda}\cdot\frac{s+1}{s}})(\frac{s\lambda}{2v(s+1)} - \frac{1}{k_{\text{load}} + \frac{2v}{\lambda}\cdot\frac{s+1}{s}})+ \\ (1-\alpha)(e^{-\frac{2L}{\lambda}} - e^{-\frac{4L}{\lambda}})(\frac{\lambda}{4v} - \frac{1}{k_{\text{load}} + \frac{4v}{\lambda}})] \end{aligned} \tag{108}$$

$$B_{2,\text{long-lived}}(L) = A_{\text{long-lived}}(L) - B_{1,\text{long-lived}}(L) \tag{109}$$

$P_{\text{synapsis,long-lived}}$ can then be computed as:

$$P_{\text{synapsis,long-lived}} = P_{\text{end-joining}|\text{constrained,long-lived}} \cdot P_{\text{constrained,long-lived}} \tag{110}$$

Eq.(110) can accurately predict the synapsis efficiency with a small fraction of long-lived LEFs, validating our mechanistic explanation of a small fraction of long-lived LEFs facilitating synapsis by improving the chance of DSB happening inside a loop and increasing the lifetime of constraining LEFs:

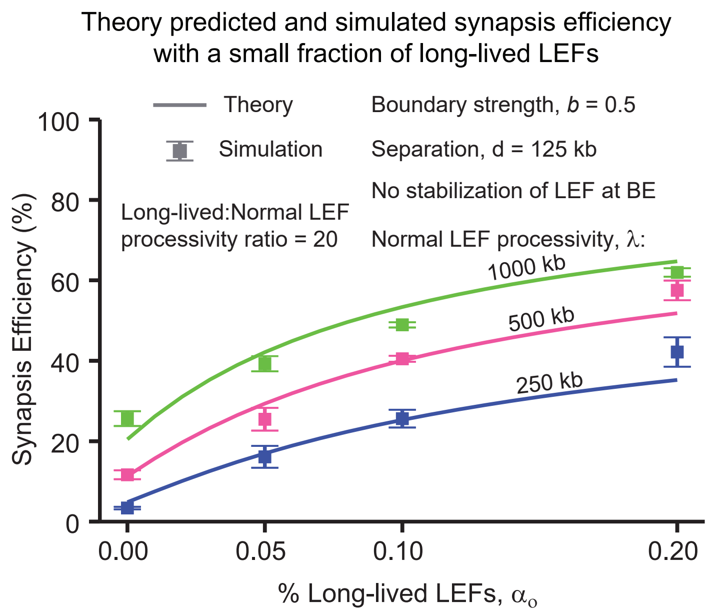

Theory predicts the synapsis efficiency with a small fraction of long-lived LEFs. Same as the bottom panel in main text **Fig. 4b**. The error bars represent the standard error of mean, n = 3 independent 1D simulations, with 218 DSB events per simulation.

1.4.3 Synapsis with LEFs stabilized by DSB ends

Mechanisms that stabilize gap-bridging LEFs will increase the likelihood of simultaneous gap-bridging events on both sides of the DSB. One such mechanism is the stabilization of LEFs by DSB ends which affects the lifetime, G, of the subpopulation the gap-bridging LEFs that have come into contact with the DSB end. Let, r, be the fold increase in G. We note that G was introduced in Section 1.3.4, to account for the lifetime of gap-bridging LEFs that have already finished gap bridging - therefore, as a first order approximation to simplify our calculations, r only affects gap-bridging LEFs that have fully bridge $L_{1^{st}}$, but does not help promote the initial bridging process. The modified PDF of the gap-bridging LEF lifetime can be written as:

$$f_{G,\text{DSBstabilized}}(g) = \frac{1}{\langle \tau_{g,\text{DSBstabilized}} \rangle} \exp\left(-\frac{g}{\langle \tau_{g,\text{DSBstabilized}} \rangle} \right) \tag{111}$$

in which:

$$\langle \tau_{g,\text{DSBstabilized}} \rangle = \frac{r\lambda}{2v} \tag{112}$$

The modified f_G updates Eq.(67) and Eqs.(69)-(70) to the following:

$$\begin{aligned} P_{\text{end-joining}|\text{constrained,DSBstabilized}} = \frac{1}{l}\int_0^l &[1 - e^{-\frac{l}{d}} + e^{-\frac{l}{d}} A(L_{1^{st}})] \cdot \{B_{1,\text{DSBstabilized}}(l - L_{1^{st}}) / \\ &[1 - B_{2,\text{DSBstabilized}}(l - L_{1^{st}}) \cdot B_{2,\text{DSBstabilized}}(L_{1^{st}})] + B_{1,\text{DSBstabilized}}(L_{1^{st}}) \cdot \\ &B_{2,\text{DSBstabilized}}(l - L_{1^{st}}) / [1 - B_{2,\text{DSBstabilized}}(l - L_{1^{st}}) \cdot B_{2,\text{DSBstabilized}}(L_{1^{st}})]\} dL_{1^{st}} \end{aligned} \tag{113}$$

$$B_{1,\text{DSBstabilized}}(L) = \frac{2v}{L}\left(e^{-\frac{L}{\lambda}\cdot\frac{r+1}{r}} - e^{-\frac{2L}{\lambda}\cdot\frac{r+1}{r}}\right)\left(\frac{r\lambda}{2v(r+1)} - \frac{1}{k_{\text{load}} + \frac{2v}{\lambda}\cdot\frac{r+1}{r}}\right) \tag{114}$$

$$B_{2,\text{DSBstabilized}}(L) = A(L) - B_{1,\text{DSBstabilized}}(L) \tag{115}$$

Note that since DSB stabilization is a reactive mechanism that only acts post DSB occurrence, it does not change the probability of DSB occurring in loops. Thus $P_{\text{synapsis,DSBstabilized}}$ can then be computed as:

$$P_{\text{synapsis,DSBstabilized}} = P_{\text{end-joining}|\text{constrained,DSBstabilized}} \cdot P_{\text{constrained,DSBstabilized}} \tag{116}$$

Eq.(116) can accurately predict the synapsis efficiency with DSB stabilization, validating our mechanistic explanation of DSB stabilization facilitating synapsis by increasing chance of simultaneous gap-bridging on both sides of the DSB through prolonged lifetime of gap-bridging LEFs:

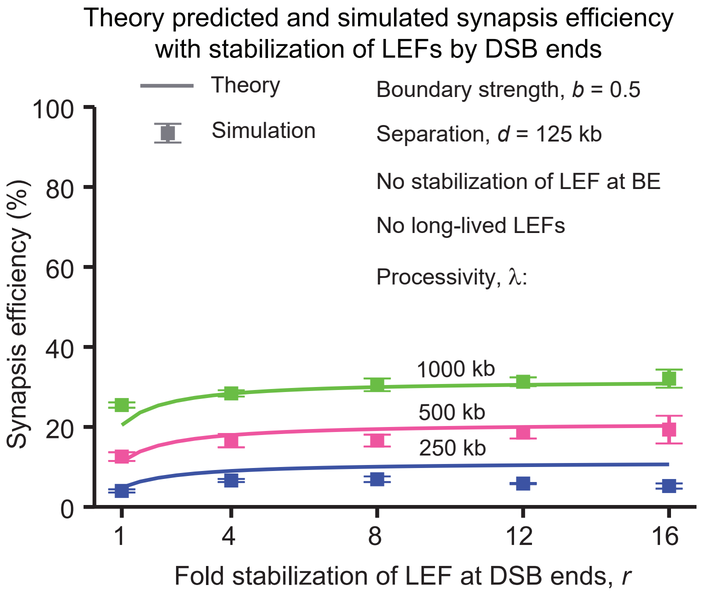

Theory predicts the synapsis efficiency with DSB stabilization. Same as the bottom panel in main text **Fig. 4c**. The error bars represent the standard error of mean, n = 3 independent 1D simulations, with 218 DSB events per simulation.

1.4.4 Synapsis with targeted loading of LEFs to DSB ends

One mechanism that can reduce τ_{loading} and thereby increase $P_{\text{end-joining}|\text{constrained}}$ is targeted loading of LEFs to the DSB site. Let, F, be the targeted loading factor (i.e., the fold increase in loading probability at the DSB compared with anywhere else in the genome). Let, U, be the average distance between two adjacent DSBs in kb, and in all simulations of the paper, we use $U = 10000 \text{ kb}$, which corresponds to one DSB occurring every 10 Mb. The PDF of the loading time X can then be modified as the following:

$$f_{X,\text{targeted}}(x) = k_{\text{load,targeted}} \exp\left(-k_{\text{load,targeted}}x\right) = \frac{1}{\langle\tau_{\text{load,targeted}}\rangle} \exp\left(-\frac{x}{\langle\tau_{\text{load,targeted}}\rangle}\right) \tag{117}$$

where:

$$k_{\text{load,targeted}} = \frac{2v}{\lambda} \cdot \frac{(F + L - 1) \cdot U}{(2F + U - 2) \cdot d} \tag{118}$$

The modified $\langle\tau_{\text{load}}\rangle$ updates Eqs.(67)-(70) to the following:

$$\begin{aligned} P_{\text{end-joining}|\text{constrained,targeted}} = \frac{1}{l}\int_0^l [1 - e^{-\frac{l}{d}} + e^{-\frac{l}{d}} A_{\text{targeted}}(L_{1^{st}})] \cdot \{B_{1,\text{targeted}}(l - L_{1^{st}})/ \\ [1 - B_{2,\text{targeted}}(l - L_{1^{st}}) \cdot B_{2,\text{targeted}}(L_{1^{st}})] + B_{1,\text{targeted}}(L_{1^{st}}) \cdot \\ B_{2,\text{targeted}}(l - L_{1^{st}})/[1 - B_{2,\text{targeted}}(l - L_{1^{st}}) \cdot B_{2,\text{targeted}}(L_{1^{st}})]\} dL_{1^{st}} \end{aligned} \tag{119}$$

$$A_{\text{targeted}}(L) = \frac{2v}{L}(e^{-\frac{L}{\lambda}} - e^{-\frac{2L}{\lambda}})(\frac{\lambda}{2v} - \frac{1}{k_{\text{load,targeted}} + \frac{2v}{\lambda}}) \tag{120}$$

$$B_{1,\text{targeted}}(L) = \frac{2v}{L}(e^{-\frac{2L}{\lambda}} - e^{-\frac{4L}{\lambda}})(\frac{\lambda}{4v} - \frac{1}{k_{\text{load,targeted}} + \frac{4v}{\lambda}}) \tag{121}$$

$$B_{2,\text{targeted}}(L) = A_{\text{targeted}}(L) - B_{1,\text{targeted}}(L) \tag{122}$$

Like DSB stabilization, targeted loading is also a reactive mechanism that only acts post DSB occurrence, it does not change the probability of DSB occurring in loops. Thus $P_{\text{synapsis,targeted}}$ can then be computed as:

$$P_{\text{synapsis,targeted}} = P_{\text{end-joining}|\text{constrained}} \cdot P_{\text{constrained,targeted}} \tag{123}$$

Eq.(123) can accurately predict the synapsis efficiency with targeted loading of LEFs at DSB, validating our mechanistic explanation of targeted loading facilitating synapsis by reducing τ_{loading}:

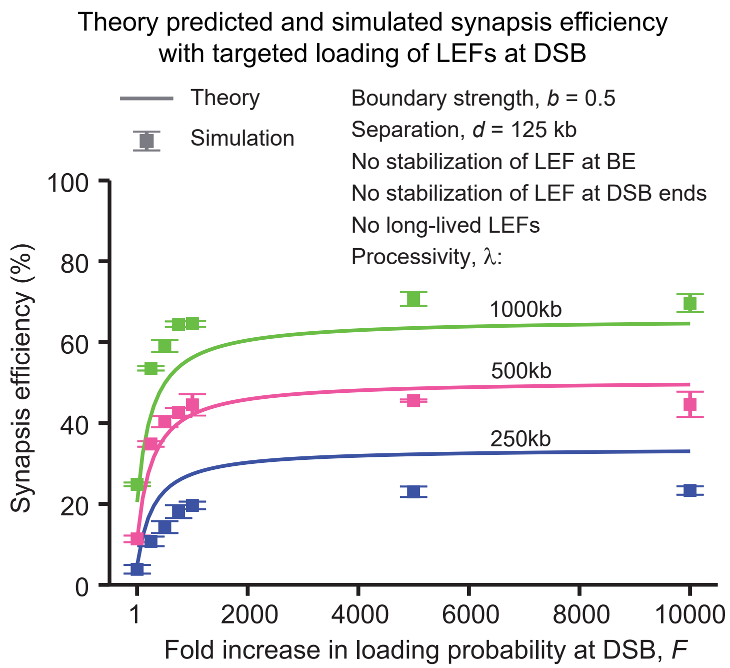

Theory predicts the synapsis efficiency with targeted loading of LEFs at DSB. Same as main text **Fig. 5b**. The error bars represent the standard error of mean, n = 3 independent 1D simulations, with 218 DSB events per simulation.

1.4.5 Synapsis with all four mechanisms combined

Now that we have explored how individual mechanisms facilitate synapsis, we can determine their combined effects. Only BE stabilization and the presence of long-lived LEFs affect $P_{\mathrm{constrained}}$, as both DSB stabilization and targeted loading are reactive mechanisms. To determine the combined effect of BE stabilization and the presence of long-lived on $P_{\mathrm{constrained}}$ without further complicating the mathematical form of the solutions and to maximally utilize the framework developed above, we first replace λ in Eq.(80) with the weighted average $\lambda_{\text{long-lived}}$ defined in Eq.(96) to obtain an updated expression of $\frac{[\mathrm{BE\text{-}LEF}]}{[\mathrm{LEF_o}]}$ and $\frac{[\mathrm{LEF}]}{[\mathrm{LEF_o}]}$:

$$\frac{[\mathrm{BE\text{-}LEF}]}{[\mathrm{LEF_o}]}_{\mathrm{combined}} = \begin{cases} \frac{d}{2\bar{D}} + \frac{1}{2} + \frac{d}{bw\lambda_{\text{long-lived}}} - \sqrt{(\frac{d}{2\bar{D}} + \frac{1}{2} + \frac{d}{bw\lambda_{\text{long-lived}}})^2 - \frac{d}{\bar{D}}} & \text{if } b > 0 \\ 0 & \text{if } b = 0 \end{cases} \tag{124}$$

$$\frac{[\mathrm{LEF}]}{[\mathrm{LEF_o}]}_{\mathrm{combined}} = 1 - \frac{[\mathrm{BE\text{-}LEF}]}{[\mathrm{LEF_o}]}_{\mathrm{combined}} \tag{125}$$

Then the fraction of LEFs stabilized by BEs, β, can be updated accordingly:

$$\beta_{\mathrm{combined}} = 1 - \left(1 - \frac{[\mathrm{BE\text{-}LEF}]}{[\mathrm{LEF_o}]}_{\mathrm{combined}}\right)^2 \tag{126}$$

Now we can use the following weighted average processivity to calculate the average loop size, as an approximation of the combined effect of BE stabilization and long-lived LEFs on $P_{\mathrm{constrained}}$:

$$\lambda_{\mathrm{combined}} = \beta_{\mathrm{combined}} \cdot w\lambda_{\text{long-lived}} + (1 - \beta_{\mathrm{combined}}) \cdot \lambda_{\text{long-lived}} \tag{127}$$

$$z_{\mathrm{combined}} = \log_{10}(\frac{\lambda_{\mathrm{combined}}}{d}) \tag{128}$$

$$\begin{aligned} a_{\mathrm{combined}} = & -0.08238 + 0.7258 z_{\mathrm{combined}} - 0.2514 z_{\mathrm{combined}}^2 \\ & - 0.003995 z_{\mathrm{combined}}^3 + 0.03445 z_{\mathrm{combined}}^4 - 0.01077 z_{\mathrm{combined}}^5 \\ & + 0.001371 z_{\mathrm{combined}}^6 - 6.472 \cdot 10^{-5} z_{\mathrm{combined}}^7 \end{aligned} \tag{129}$$

$$l_{\mathrm{combined}} = 10^{a_{\mathrm{combined}}} \cdot d \tag{130}$$

Eqs.(124)-(125) and Eq.(130) in turn update the probability of DSB happening in loops defined in Eq.(16) to the following:

$$P_{\mathrm{constrained,combined}} =$$

$$\begin{cases} (1 - e^{-\frac{l_{\mathrm{combined}}}{d}})/(1 + \frac{[\mathrm{BE\text{-}LEF}]}{[\mathrm{LEF_o}]}_{\mathrm{combined}}(1 - e^{-\frac{d}{l_{\mathrm{combined}}}}) \cdot \\ (\frac{d}{l_{\mathrm{combined}}} - 1 + (\frac{d}{l_{\mathrm{combined}}} + 1)e^{-\frac{d}{l_{\mathrm{combined}}}})) & \text{if } \frac{d}{2} \leq \bar{D} \\ (1 - e^{-\frac{l_{\mathrm{combined}}}{d}})/(1 + \frac{[\mathrm{BE\text{-}LEF}]}{[\mathrm{LEF_o}]}_{\mathrm{combined}} \frac{[\mathrm{LEF}]}{[\mathrm{LEF_o}]}_{\mathrm{combined}}(1 - e^{-\frac{d}{l_{\mathrm{combined}}}}) \cdot \\ (\frac{d}{l_{\mathrm{combined}}} - 1 + (\frac{d}{l_{\mathrm{combined}}} + 1)e^{-\frac{d}{l_{\mathrm{combined}}}}) + (\frac{[\mathrm{BE\text{-}LEF}]}{[\mathrm{LEF_o}]}_{\mathrm{combined}})^2 \frac{\bar{D}}{l_{\mathrm{combined}}}) & \text{if } \frac{d}{2} > \bar{D} \end{cases} \tag{131}$$

For simplicity, we neglect the effects of BE and DSB stabilization, and long-lived LEFs on the loading time as an approximation:

$$k_{\mathrm{load,combined}} = k_{\mathrm{load,targeted}} \tag{132}$$

$$f_{X,\mathrm{combined}}(x) = k_{\mathrm{load,combined}} \exp(-k_{\mathrm{load,combined}} x) = k_{\mathrm{load,targeted}} \exp(-k_{\mathrm{load,targeted}} x) \tag{133}$$

We approximated the combined effect of long-lived LEFs and BE stabilization on the constraining LEF lifetime distribution as the following:

$$f_{C,\mathrm{combined}}(c) = (1 - \alpha) \cdot \frac{1}{\langle \tau_{c1} \rangle} \exp\left(-\frac{c}{\langle \tau_{c1} \rangle}\right) + \alpha \cdot \frac{1}{\langle \tau_{c2} \rangle} \exp\left(-\frac{c}{\langle \tau_{c2} \rangle}\right) \tag{134}$$

in which:

$$\langle \tau_{c1} \rangle = \frac{w\lambda}{2v} \tag{135}$$

$$\langle \tau_{c2} \rangle = \frac{ws\lambda}{2v} \tag{136}$$

As an approximation, we neglect the scenario of long-lived LEFs acting as gap-bridging LEFs, since long-lived LEFs are much less likely to be loaded at a DSB to function as gap-bridging LEFs given their slow dynamics. Thus the PDF of the gap-bridging LEF lifetime remains the same as Eq.(111):

$$f_{G,\text{combined}}(g) = \frac{1}{\langle \tau_{g,\text{DSBstabilized}} \rangle} \exp\left(-\frac{g}{\langle \tau_{g,\text{DSBstabilized}} \rangle}\right) \tag{137}$$

Subsequently, with all four mechanisms combined, Eqs.(67)-(70) are modified to the following:

$$\begin{aligned} P_{\text{end-joining}|\text{constrained,combined}} = \frac{1}{l}\int_0^l & [1 - e^{-\frac{l}{d}} + e^{-\frac{l}{d}} A_{\text{combined}}(L_{1^{st}})] \cdot \{B_{1,\text{combined}}(l - L_{1^{st}})/ \\ & [1 - B_{2,\text{combined}}(l - L_{1^{st}}) \cdot B_{2,\text{combined}}(L_{1^{st}})] + B_{1,\text{combined}}(L_{1^{st}}) \cdot \\ & B_{2,\text{combined}}(l - L_{1^{st}})/[1 - B_{2,\text{combined}}(l - L_{1^{st}}) \cdot B_{2,\text{combined}}(L_{1^{st}})]\} dL_{1^{st}} \end{aligned} \tag{138}$$

$$\begin{aligned} A_{\text{combined}}(L) = \frac{2v}{L}[& \alpha(e^{-\frac{L}{ws\lambda}} - e^{-\frac{2L}{ws\lambda}})(\frac{ws\lambda}{2v} - \frac{1}{k_{\text{load,targeted}} + \frac{2v}{ws\lambda}}) + \\ & (1-\alpha)(e^{-\frac{L}{w\lambda}} - e^{-\frac{2L}{w\lambda}})(\frac{w\lambda}{2v} - \frac{1}{k_{\text{load,targeted}} + \frac{2v}{w\lambda}})] \end{aligned} \tag{139}$$

$$\begin{aligned} B_{1,\text{combined}}(L) = \frac{2v}{L}[& \alpha(e^{-\frac{L}{\lambda}\cdot\frac{ws+r}{wsr}} - e^{-\frac{2L}{\lambda}\cdot\frac{ws+r}{wsr}})(\frac{wsr\lambda}{2v(ws+r)} - \frac{1}{k_{\text{load,targeted}} + \frac{2v}{\lambda}\cdot\frac{ws+r}{wsr}}) + \\ & (1-\alpha)(e^{-\frac{L}{\lambda}\cdot\frac{w+r}{wr}} - e^{-\frac{2L}{\lambda}\cdot\frac{w+r}{wr}})(\frac{wr\lambda}{2v(w+r)} - \frac{1}{k_{\text{load,targeted}} + \frac{2v}{\lambda}\cdot\frac{w+r}{wr}})] \end{aligned} \tag{140}$$

$$B_{2,\text{combined}}(L) = A_{\text{combined}}(L) - B_{1,\text{combined}}(L) \tag{141}$$

$P_{\text{end-joining}|\text{constrained,combined}}$ can now be calculated by substituting Eqs.(139)-(141) into Eq.(138) and performing numerical integration (see examples in the Mathematica notebooks in the GitHub repository).

$P_{\text{synapsis,combined}}$ can then be computed as:

$$\boxed{P_{\text{synapsis,combined}} = P_{\text{end-joining}|\text{constrained,combined}} \cdot P_{\text{constrained,combined}}} \tag{142}$$

Eq.(142) can predict the synapsis efficiency with all four additional mechanisms combined with reasonable accuracy, supporting our mechanistic explanation of how these mechanisms come together to facilitate DSB end synapsis:

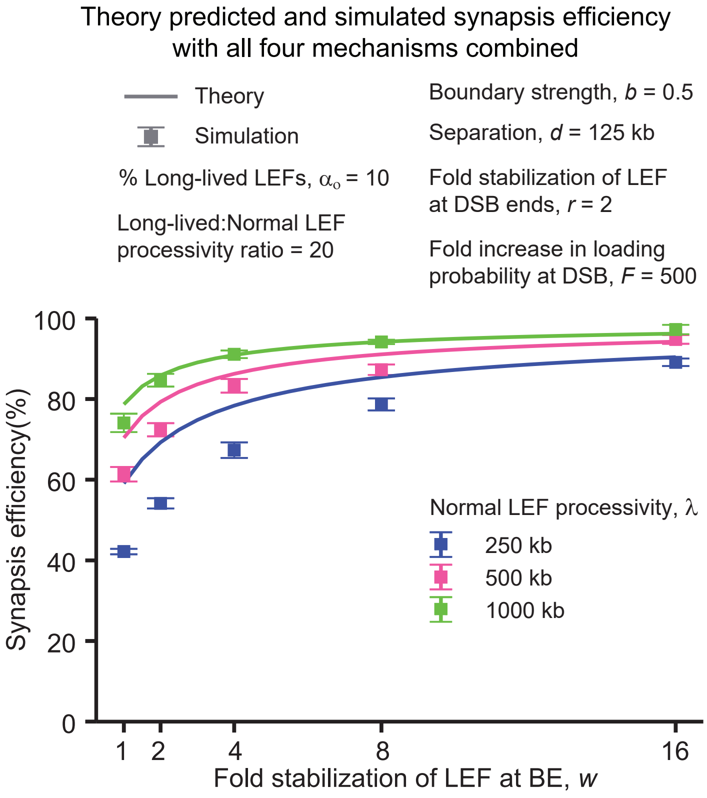

Theory predicts the synapsis efficiency with all four additional mechanisms combined. The error bars represent the standard error of mean, n = 3 independent 1D simulations, with 218 DSB events per simulation.

1.5 Simplified expression for the probability of synapsis with four additional mechanisms

We can derive the simplified expression for the probability of synapsis with all four mechanisms combined.

For $P_{\text{constrain,combined}}$, we can first apply the same linear approximation (tangent line approximation) of $\frac{[\text{BE-LEF}]}{[\text{LEF}_\text{o}]}$ used to obtain Eq.(31):

$$P_{\text{constrained,combined}} \approx$$

$$\begin{cases} (1-e^{-\frac{l_{\text{combined}}}{d}})/(1+\frac{(1-e^{-\frac{d}{l_{\text{combined}}}})(\frac{d}{l_{\text{combined}}}-1+(\frac{d}{l_{\text{combined}}}+1)e^{-\frac{d}{l_{\text{combined}}}})}{1+\frac{\bar{D}}{d}+\frac{2\bar{D}}{bw\lambda_{\text{long-lived}}}}) & \text{if } \frac{d}{2} \leq \bar{D} \\ (1-e^{-\frac{l_{\text{combined}}}{d}})/(1+(\frac{(\frac{\bar{D}}{d}+\frac{2\bar{D}}{bw\lambda_{\text{long-lived}}})(1-e^{-\frac{d}{l_{\text{combined}}}})(\frac{d}{l_{\text{combined}}}-1+(\frac{d}{l_{\text{combined}}}+1)e^{-\frac{d}{l_{\text{combined}}}})+\frac{\bar{D}}{l_{\text{combined}}}}{(1+\frac{\bar{D}}{d}+\frac{2\bar{D}}{bw\lambda_{\text{long-lived}}})^2})) & \text{if } \frac{d}{2} > \bar{D} \end{cases} \tag{143}$$

For $P_{\text{end-joining}|\text{constrained,combined}}$, similar to above, we can simplify by considering the scenario where gaps are bridged upon the first try (before the first gap-bridging LEF falls off), with gap length $L_{1^{st}}$ and $L_{2^{nd}}$ on two sides of the DSB. We assume no gap-bridging LEFs are present within the constraining LEF at the time of DSB occurrence. Then $P_{\text{end-joining}|\text{constrained,combined}}$ can be approximated as the following:

$$\begin{aligned}
&P_{\text{end-joining}|\text{constrained,combined}} \approx A_{\text{combined}}(\frac{l}{2}) \cdot B_{1,\text{combined}}(\frac{l}{2}) \\
&= \frac{v}{l}[\alpha(e^{-\frac{l}{2ws\lambda}} - e^{-\frac{l}{ws\lambda}})(\frac{ws\lambda}{2v} - \frac{1}{k_{\text{load,targeted}} + \frac{2v}{ws\lambda}})+ \\
&(1-\alpha)(e^{-\frac{l}{2w\lambda}} - e^{-\frac{l}{w\lambda}})(\frac{w\lambda}{2v} - \frac{1}{k_{\text{load,targeted}} + \frac{2v}{w\lambda}})]\cdot \\
&\frac{v}{l}[\alpha(e^{-\frac{l}{2\lambda}\cdot\frac{ws+r}{wsr}} - e^{-\frac{l}{\lambda}\cdot\frac{ws+r}{wsr}})(\frac{wsr\lambda}{2v(ws+r)} - \frac{1}{k_{\text{load,targeted}} + \frac{2v}{\lambda}\cdot\frac{ws+r}{wsr}})+ \\
&(1-\alpha)(e^{-\frac{l}{2\lambda}\cdot\frac{w+r}{wr}} - e^{-\frac{l}{\lambda}\cdot\frac{w+r}{wr}})(\frac{wr\lambda}{2v(w+r)} - \frac{1}{k_{\text{load,targeted}} + \frac{2v}{\lambda}\cdot\frac{w+r}{wr}})] \\
&= [\frac{\alpha(ws\tau_{\text{constrained}})^2}{\tau_{\text{extrusion}}(\tau_{\text{loading,targeted}} + ws\tau_{\text{constrained}})}(e^{-\frac{\tau_{\text{extrusion}}}{ws\tau_{\text{constrained}}}} - e^{-\frac{2\tau_{\text{extrusion}}}{ws\tau_{\text{constrained}}}})+ \\
&\frac{(1-\alpha)(w\tau_{\text{constrained}})^2}{\tau_{\text{extrusion}}(\tau_{\text{loading,targeted}} + w\tau_{\text{constrained}})}(e^{-\frac{\tau_{\text{extrusion}}}{w\tau_{\text{constrained}}}} - e^{-\frac{2\tau_{\text{extrusion}}}{w\tau_{\text{constrained}}}})]\cdot \\
&[\frac{\alpha(\frac{wsr}{ws+r}\tau_{\text{constrained}})^2}{\tau_{\text{extrusion}}(\tau_{\text{loading,targeted}} + \frac{wsr}{ws+r}\tau_{\text{constrained}})}(e^{-\frac{\tau_{\text{extrusion}}}{\frac{wsr}{ws+r}\tau_{\text{constrained}}}} - e^{-\frac{2\tau_{\text{extrusion}}}{\frac{wsr}{ws+r}\tau_{\text{constrained}}}})+ \\
&\frac{(1-\alpha)(\frac{wr}{w+r}\tau_{\text{constrained}})^2}{\tau_{\text{extrusion}}(\tau_{\text{loading,targeted}} + \frac{wr}{w+r}\tau_{\text{constrained}})}(e^{-\frac{\tau_{\text{extrusion}}}{\frac{wr}{w+r}\tau_{\text{constrained}}}} - e^{-\frac{2\tau_{\text{extrusion}}}{\frac{wr}{w+r}\tau_{\text{constrained}}}})] \\
&= [\frac{\alpha f(\frac{\tau_{\text{extrusion}}}{ws\tau_{\text{constrained}}})}{1+\frac{\tau_{\text{loading,targeted}}}{ws\tau_{\text{constrained}}}} + \frac{(1-\alpha)f(\frac{\tau_{\text{extrusion}}}{w\tau_{\text{constrained}}})}{1+\frac{\tau_{\text{loading,targeted}}}{w\tau_{\text{constrained}}}}]\cdot[\frac{\alpha f(\frac{\tau_{\text{extrusion}}}{\frac{wsr}{ws+r}\tau_{\text{constrained}}})}{1+\frac{\tau_{\text{loading,targeted}}}{\frac{wsr}{ws+r}\tau_{\text{constrained}}}} + \frac{(1-\alpha)f(\frac{\tau_{\text{extrusion}}}{\frac{wr}{w+r}\tau_{\text{constrained}}})}{1+\frac{\tau_{\text{loading,targeted}}}{\frac{wr}{w+r}\tau_{\text{constrained}}}}]
\end{aligned} \tag{144}$$

where:

$$\begin{aligned}
\tau_{\text{loading,targeted}} &= \frac{1}{k_{\text{load,targeted}}} \\
&= \frac{\lambda}{2v}\cdot\frac{(2F+U-2)\cdot d}{(F+\frac{l}{2}-1)\cdot U}
\end{aligned} \tag{145}$$

Now we can write the simplified expression for $P_{\text{synapsis, combined}}$:

$$P_{\text{synapsis,combined}} \approx$$

$$\begin{cases} (1-e^{-\frac{l_{\text{combined}}}{d}})/(1+\frac{(1-e^{-\frac{d}{l_{\text{combined}}}})(\frac{d}{l_{\text{combined}}}-1+(\frac{d}{l_{\text{combined}}}+1)e^{-\frac{d}{l_{\text{combined}}}})}{1+\frac{\bar{D}}{d}+\frac{2\bar{D}}{bw\lambda_{\text{long-lived}}}})\cdot \\ [\frac{\alpha f(\frac{\tau_{\text{extrusion}}}{ws\tau_{\text{constrained}}})}{1+\frac{\tau_{\text{loading,targeted}}}{ws\tau_{\text{constrained}}}} + \frac{(1-\alpha)f(\frac{\tau_{\text{extrusion}}}{w\tau_{\text{constrained}}})}{1+\frac{\tau_{\text{loading,targeted}}}{w\tau_{\text{constrained}}}}]\cdot[\frac{\alpha f(\frac{\tau_{\text{extrusion}}}{\frac{wsr}{ws+r}\tau_{\text{constrained}}})}{1+\frac{\tau_{\text{loading,targeted}}}{\frac{wsr}{ws+r}\tau_{\text{constrained}}}} + \frac{(1-\alpha)f(\frac{\tau_{\text{extrusion}}}{\frac{wr}{w+r}\tau_{\text{constrained}}})}{1+\frac{\tau_{\text{loading,targeted}}}{\frac{wr}{w+r}\tau_{\text{constrained}}}}] & \text{if } \frac{d}{2} \leq \bar{D} \\ (1-e^{-\frac{l_{\text{combined}}}{d}})/(1+(\frac{(\frac{\bar{D}}{d}+\frac{2\bar{D}}{bw\lambda_{\text{long-lived}}})(1-e^{-\frac{d}{l_{\text{combined}}}})(\frac{d}{l_{\text{combined}}}-1+(\frac{d}{l_{\text{combined}}}+1)e^{-\frac{d}{l_{\text{combined}}}})+\frac{\bar{D}}{l_{\text{combined}}}}{(1+\frac{\bar{D}}{d}+\frac{2\bar{D}}{bw\lambda_{\text{long-lived}}})^2}))\cdot \\ [\frac{\alpha f(\frac{\tau_{\text{extrusion}}}{ws\tau_{\text{constrained}}})}{1+\frac{\tau_{\text{loading,targeted}}}{ws\tau_{\text{constrained}}}} + \frac{(1-\alpha)f(\frac{\tau_{\text{extrusion}}}{w\tau_{\text{constrained}}})}{1+\frac{\tau_{\text{loading,targeted}}}{w\tau_{\text{constrained}}}}]\cdot[\frac{\alpha f(\frac{\tau_{\text{extrusion}}}{\frac{wsr}{ws+r}\tau_{\text{constrained}}})}{1+\frac{\tau_{\text{loading,targeted}}}{\frac{wsr}{ws+r}\tau_{\text{constrained}}}} + \frac{(1-\alpha)f(\frac{\tau_{\text{extrusion}}}{\frac{wr}{w+r}\tau_{\text{constrained}}})}{1+\frac{\tau_{\text{loading,targeted}}}{\frac{wr}{w+r}\tau_{\text{constrained}}}}] & \text{if } \frac{d}{2} > \bar{D} \end{cases} \tag{146}$$

1.6 Two important relative timescales underpinning synapsis efficiency

Since estimates based on experimental evidence suggest that most of the interphase DNA is inside loops at any given time ([1, 3, 4]), $P_{\text{constrained,combined}}$ is likely close to 1. Thus achieving close-to-perfect synapsis efficiency hinges on $P_{\text{end-joining}|\text{constrained,combined}}$. We next ask what factors underlying $P_{\text{end-joining}|\text{constrained,combined}}$ have the most dominant impact on synapsis efficiency. Notice the recurrent terms, $\frac{\tau_{\text{loading}}}{\tau_{\text{constrained}}}$ and $\frac{\tau_{\text{extrusion}}}{\tau_{\text{constrained}}}$, with different prefactors in Eq.(144), we define the following weighted relative timescales:

$$(\frac{\tau_{\text{loading}}}{\tau_{\text{constrained}}})_{\text{weighted}} = \{[\frac{\alpha}{ws}\frac{\tau_{\text{loading,targeted}}}{\tau_{\text{constrained}}} + \frac{1-\alpha}{w}\frac{\tau_{\text{loading,targeted}}}{\tau_{\text{constrained}}}] \cdot [\alpha(\frac{1}{ws}+\frac{1}{r})\frac{\tau_{\text{loading,targeted}}}{\tau_{\text{constrained}}} + (1-\alpha)(\frac{1}{w}+\frac{1}{r})\frac{\tau_{\text{loading,targeted}}}{\tau_{\text{constrained}}}]\}^{0.5} \tag{147}$$

$$(\frac{\tau_{\text{extrusion}}}{\tau_{\text{constrained}}})_{\text{weighted}} = \{[\frac{\alpha}{ws}\frac{\tau_{\text{extrusion}}}{\tau_{\text{constrained}}} + \frac{1-\alpha}{w}\frac{\tau_{\text{extrusion}}}{\tau_{\text{constrained}}}] \cdot [\alpha(\frac{1}{ws}+\frac{1}{r})\frac{\tau_{\text{extrusion}}}{\tau_{\text{constrained}}} + (1-\alpha)(\frac{1}{w}+\frac{1}{r})\frac{\tau_{\text{extrusion}}}{\tau_{\text{constrained}}}]\}^{0.5} \tag{148}$$

On top of our theoretical framework, we also used 1D simulations to determine whether all four mechanisms could combine to improve synapsis efficiency. We added processivity/separation as an additional dimension, resulting in a 5-dimensional parameter scan with 768 different parameter combinations (see main text **Fig. 6a**). The two weighted relative timescales in Eqs.(147)-(148) can effectively separate the 5-dimensional parameter scan simulation data points based on synapsis efficiency, suggesting lowering the two relative timescales is key to improving synapsis efficiency:

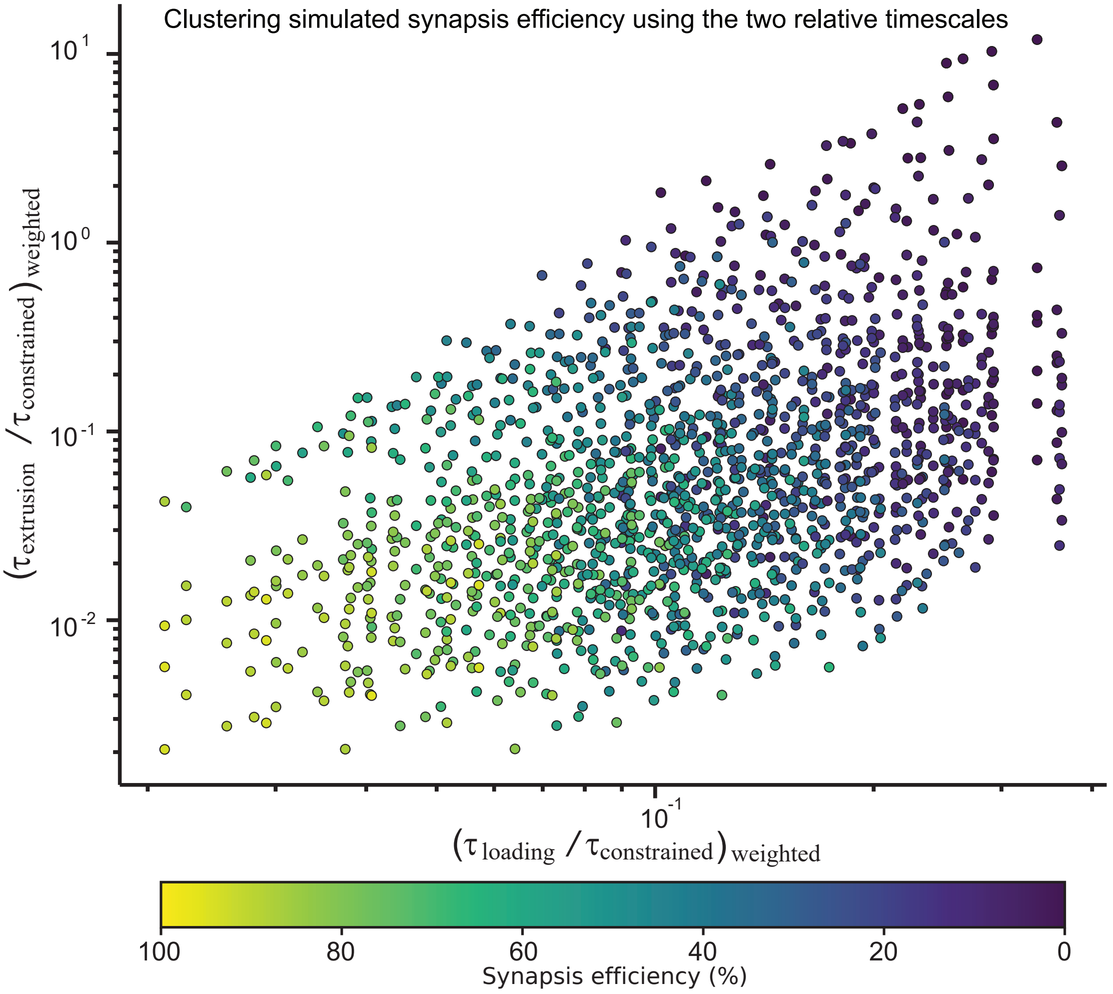

The two relative timescales can effectively cluster simulation data points according to their synapsis efficiency. Same as main text **Fig. 6b**. The color of each data point shows the average synapsis efficiency of n = 3 independent 1D simulations for a given parameter combination, with 218 DSB events per simulation. The two relative timescales for each data point are calculated using Eqs.(147)-(148) based on the input parameters.

1.7 Limitations of our analytical theory and model

Despite the close agreement between our theory prediction of synapsis efficiency and the simulation results in the parameter space bounded by experimental estimates (see **Supplementary Note 2**), it is worth pointing out several important assumptions and approximations made to simplify the mathematical form of the analytical solution:

1. We assume the prolonged LEF lifetime due to additional mechanisms (BE and DSB stabilization, and long-lived LEFs) does not affect the loading time Y. While the accuracy of predictions is largely unaffected by this assumption when $\lambda > d$ as shown above, the assumption no longer holds when $\lambda \leq d$ as the prolonged lifetime for a fraction of LEFs reduces the pool of dynamic LEFs that can be quickly loaded at DSB, leading to overestimation of synapsis efficiency:

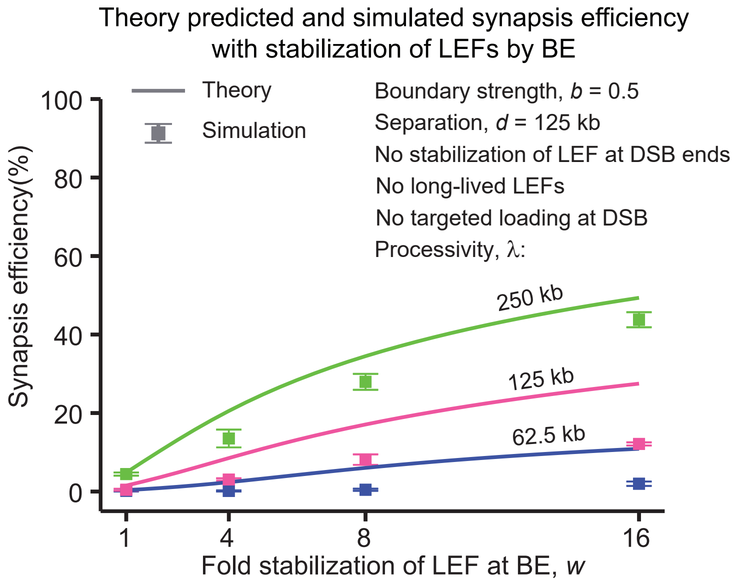

Theory overestimates the synapsis efficiency when $\lambda \leq d$. The error bars represent the standard error of mean, n = 3 independent 1D simulations, with 218 DSB events per simulation.

2. We assume that only one gap-bridging LEF is bridging the gap on each side of the DSB for the calculation of the first-passage time T. This assumption no longer holds when $\lambda \gg d$ and leads to underestimation of synapsis efficiency (top right corner of the heatmaps in main text **Fig. 3b**).

3. We assume if one or more gap-bridging LEFs are present within the constraining LEF at the time of DSB occurrence, one of the two gaps is bridged already. While a seemingly crude approximation to account for the pre-existing gap-bridging LEFs' impact on synapsis, this assumption does not significantly affect the accuracy of theory prediction.

Perhaps the most significant limitation of our theory is that we neglect the effect of passive 3D diffusion. While we estimate the time it takes for 3D diffusion alone to bring two DSB ends back into proximity is likely too long to be consistent with the synapsis timescale, 3D diffusion likely acts concurrently and synergistically with loop extrusion to further improve synapsis efficiency. When the two DSB ends are brought close enough by loop extrusion, the chance of the two DSB ends randomly encountering each other through passive diffusion also greatly increases. Therefore, the synapsis efficiency predicted by our theory likely delineates the lower bound of the physiological synapsis efficiency.

Finally, we have also made several biological assumptions that abstract the complex synapsis process into a simplified picture more tractable for theoretical analysis:

1. We assume uniform loading probability of LEFs across the genome aside from DSB ends. While it remains unclear to what extent cohesins show preferential loading to certain genomic regions, previous work suggests that cohesin loading may not be uniform across the genome [5–7].

2. We assume uniform LEF extrusion speed throughout the genome and that only BEs may stall LEF extrusion, while it has been shown that other non-BE elements such as RNA polymerases could also act as partial extrusion barriers [5, 8, 9].

3. We assume a uniform probability of DSB formation throughout the genome, while evidence suggests certain sites such as binding sites for CTCF and cohesin are more prone to generate DSBs [10–14]. Since DSB sites close to CTCF likely benefit more from BE stabilization to improve synapsis efficiency, and DSB sites close to cohesin binding sites are more likely to be constrained by LEFs and have gap-bridging LEFs loaded in the gaps to improve synapsis efficiency, our theory might underestimate the physiological synapsis efficiency by assuming a uniform probability of DSB formation.

1.8 Conclusion

In conclusion, we built a probabilistic theoretical model with a relatively simple mathematical form, that can predict the simulated synapsis efficiency in the parameter regime with $\lambda > d$ with a fairly good accuracy. Our theory highlights two important roles of loop extrusion in synapsis: LEFs can constrain DSB ends and thereby prevent them from diffusing apart, and gap-bridging LEFs can mediate synapsis by bringing the DSB ends back together. Since DSB ends can only be joined by loop extrusion if the DSB occurs inside a loop, loop coverage of the genome

is crucial to achieve high synapsis efficiency. Mechanisms that stabilize LEFs before the DSB occurs such as BE stabilization and the presence of a small fraction of long-lived LEFs could serve to improve the coverage of the genome by LEFs. Provided that the DSB ends are constrained, our theory then points to two relative timescales, $\frac{\tau_{\text{loading}}}{\tau_{\text{constrained}}}$ and $\frac{\tau_{\text{extrusion}}}{\tau_{\text{constrained}}}$ that underlie synapsis efficiency. By extending our simple theory with 4 physiologically plausible mechanisms, we show how each of the four mechanisms decreases the two relative timescales and thereby improves synapsis efficiency. The final expressions with all four mechanisms combined illustrate the synergistic effects of the mechanisms. Despite the various approximations made in deriving the theory, the relatively good agreement between our analytical theory and simulations lends credibility to our mechanistic insights obtained from our theoretical model.

Our theory can also serve to guide experimental perturbation of the DSB synapsis machinery for further validation the role of different extended mechanisms discussed above, and to explain observations of the perturbations' impact on synapsis efficiency. Our theory has direct implications for the dependence of DSB repair efficiency on genomic context. For example, our theory points to the importance of BE stabilization in efficient synapsis, which predicts reduced synapsis efficiency in genomic region that lacks BEs (CTCF binding sites etc.). Indeed, heterochromatin, which usually has lower density of bound CTCF binding sites, has been shown to be more sensitive to radiation-induced chromosomal aberrations than euchromatin in Chinese hamster cells [15], consistent with our theory prediction.

2 Supplementary Note 2

Overview

Given the large possible parameter space for individual variables considered in our simulations and modeling as well as the vast parameter combinations generated by permutation, we sought to use prior experimental estimates to generate plausible upper and lower bounds on individual parameter values. In this Supplementary Note, we detail the calculations used to justify the parameter bounds chosen in our study.

2.1 Estimation of the range of LEF separation and processivity

Two studies performed absolute quantification of CTCF and cohesin in HeLa cells [4] and mouse embryonic stem cells (mESCs) [3]. Since in simulations we assume LEFs re-load somewhere on the genome immediately after unloading, we could use the density of chromatin-bound cohesin to estimate the LEF separation, d (the inverse of LEF density).

Using fluorescence-correlation spectroscopy (FCS) and fluorescence recovery after photobleaching (FRAP), the chromatin-bound SCC1(subunit of cohesin) copy number has been estimated to be $\sim 160,000$ for HeLa cells [4]. Considering the total HeLa genome length of 7.9 Gb [4], we can estimate the cohesin spearation as the following, assuming cohesin exists as monomeric ring [16]:

$$\begin{aligned} d_{\text{Holzmann,monomer}} &= (7.9 \cdot 10^9 \text{bp})/160000 \\ &\approx 50 \text{ kb} \end{aligned} \tag{149}$$

If we assume cohesin extrudes as dimers, then [16]:

$$\begin{aligned} d_{\text{Holzmann,dimer}} &= d_{\text{Holzmann,dimer}} \cdot 2 \\ &= 100 \text{ kb} \end{aligned} \tag{150}$$

Through a combination of FCS, "in-gel" fluorescence, and flow cytometry, we previously carried out an absolute quantification of CTCF and cohesin in mESCs. We estimated the cohesin density to be 5.3 per Mb (assuming monomeric) or 2.7 per Mb (assuming dimeric) [3, 16], which correspond to the following cohesin separation:

$$\begin{aligned} d_{\text{Cattoglio,monomer}} &= 1\text{Mb}/5.3 \\ &\approx 190 \text{ kb} \end{aligned} \tag{151}$$

$$\begin{aligned} d_{\text{Cattoglio,dimer}} &= 1\text{Mb}/2.7 \\ &\approx 370 \text{ kb} \end{aligned} \tag{152}$$

Thus far, absolute quantification of cohesin has only been performed in two mammalian cell types, HeLa and mESC. As can be seen, the density varies substantially between these two cell types, and may vary even more among other cell types. It is therefore associated with significant uncertainty. Nevertheless, these two studies bound the LEF separations in the following range:

$$d \epsilon [50 \text{ kb}, 370 \text{ kb}] \tag{153}$$

Let, τ_o, be the LEF lifetime (residence time). Let v_T, be the total unobstructed LEF extrusion speed. Then for the two-sided LEFs considered in our study, $v_T = 2v$, where v is the unobstructed extrusion speed in one direction. Then LEF processivity can be computed as the product of total extrusion speed (in both directions) and the LEF lifetime:

$$\lambda = v_T \cdot \tau_o \tag{154}$$

Several single-molecule experiments for condensins and cohesins [17–21] measured the unobstructed total extrusion speed to be in the range of $v_T \epsilon [0.5 \text{ kb/s}, 2 \text{ kb/s}]$. Note that the extrusion speed estimated from *in vivo* measurement is often slower [16, 22, 23], likely due to various protein roadblocks bound to DNA including BEs like CTCF. While synapsis time calculated from our simulations is inversely proportional to extrusion speed, synapsis efficiency is independent of extrusion speed. Unless otherwise specified, we use v_T of 1 kb/s for the calculation of synapsis time.

Multiple studies have estimated cohesin's residence time which varies with cell cycle phase. Focusing on G1, we estimated cohesin's residence time to be ∼22 minutes in mESCs [24], whereas Holzmann *et al.* estimated it to be ∼13.7 minutes in HeLa cells [4]. In contrast, the residence time of condensin I was determined by FRAP to be ∼2 minutes [25]. Thus the bounds of v_T and τ_o estimated from these studies provide the following range of LEF processivity given Eq.(154):

$$\lambda \epsilon [60 \text{ kb}, 2640 \text{ kb}] \tag{155}$$

Given the estimated bounds of LEF separation and processivity in Eq.(153) and Eq.(155), to keep the processivity/separation ratio as geometric integer sequence, we use the separation list $d = [62.5, 125, 250, 500] \text{ kb}$ and the processivity list $\lambda = [62.5, 125, 250, 500, 1000] \text{ kb}$ in the simulations without additional mechanisms. To limit the parameter combinations in the the 5-dimensional parameter scan simulations, we used the separation and processivity combinations $(d, \lambda) = [(125, 62.5), (125, 125), (125, 250), (250, 125), (250, 250), (250, 500)] \text{ kb}$, so that processivity/separation ratios of 0.5,1 and 2 are examined.

2.2 Estimation of the fold increase in LEF lifetime upon stabilization by BEs

CTCF can increase cohesin's chromatin residence time by interacting with cohesin in such a way that it outcompetes cohesin interactions with WAPL, which unloads cohesin from DNA [26]. An independent study found that CTCF could also facilitate cohesin acetylation and prolong the lifetime of acetylated cohesin [27]. Cohesin contains one of the two variant STAG subunits, STAG1 or STAG2 [28]. CTCF stabilizes both cohesin-STAG1 and cohesin-STAG2, but with a lesser extent for cohesin-STAG2 [27]. Cohesin-STAG1's stable residence time, $\tau_{\text{cohesin-STAG1,stable}}$, and dynamic residence time, $\tau_{\text{cohesin-STAG1,dynamic}}$, during G1 phase have been determined by inverse fluorescence recovery after photobleaching (iFRAP) [27]:

$$\tau_{\text{cohesin-STAG1,stable}} \approx 5 \text{ hr} \tag{156}$$

$$\tau_{\text{cohesin-STAG1,dynamic}} \approx 15 \text{ min} \tag{157}$$

Since the longer lifetime of cohesin-STAG1 could be attributed to both stabilization by CTCF and acetylation of SMC3 (a subunit of cohesin) by ESCO1 and that cohesin-STAG2 is stabilized by CTCF to a lesser extent than cohesin-STAG1 [27], the ratio of the stable residence time of cohesin-STAG1 and the dynamic residence time of cohesin-STAG1 could serve as an upper bound for the fold increase in LEF lifetime due to stabilization by BEs, w:

$$w \epsilon [1, 20] \tag{158}$$

2.3 Estimation of the fraction and lifetime of long-lived LEFs

Long-lived LEFs can be conceptualized as a subpopulations of LEFs with intrinsic longer lifetime due to chemical modifications such as acetylation of the cohesin subunit SMC3. The STAG subunit of cohesin can be composed of either STAG1 or STAG2 given rise to two distinct forms of cohesin. Cohesin-STAG1 is preferentially acetylated during G1 phase, and cohesin-STAG2 contains four times less acetylated SMC3 relative to cohesin-STAG1 [27]. About $\sim 30 - 50\%$ of cohesin-STAG1 is stably bound to chromatin in G1 [24, 27, 29]. The relative levels of STAG1 and STAG2 varies substantially between cell types [30]. Cohesin-STAG1 constitutes about about 25% of total cohesin in HeLa cell [4], about 33% of total cohesins in immortalized mouse embryonic fibroblasts (iMEFs) [31], and about 55% in normal human bronchial epithelial cells (NHBE) [32], with the rest being cohesin-STAG2.

Taken together, if we assume all the stably bound cohesin-STAG1 is acetylated, up to ∼30% of all cohesin is then acetylated, which we use as an upper bound for the fraction of long-lived LEFs, α_o, since factors other than chemical modifications such as stabilization by CTCF could also contribute to the stably bound cohesin's longer lifetime [27]:

$$\alpha_o \epsilon [0, 30\%] \tag{159}$$

Stably bound SCC1 exhibits about 50 fold increase in lifetime compared with the dynamic fraction of SCC1 [27]. Thus we can bound the fold increase in long-lived LEFs' lifetime compared with normal LEFs:

$$s\epsilon[1, 50] \tag{160}$$

To limit the number of free variables, we use an intermediate value $s = 20$ throughout our study.

2.4 Estimation of the fold increase in LEF lifetime upon stabilization by DSB ends

The ATM kinase at DSB ends is hypothesized to phosphorylate cohesin, thereby increasing cohesin's lifetime [33]. When cohesin is stabilized by ATM kinase at DSB ends, the higher processivity means that there is higher probability that cohesin can extrude all the way to adjacent BEs. About 1.5 fold enrichment of SCC1, a subunit of cohesin, at BEs was observed in DSB-containing TADs relative to SCC1 count prior to DSB occurrence [33]. We performed simulations with different fold stabilization of LEF at DSB ends, and found the ∼1.5 fold enrichment of SCC1 at BEs in DSB-containing TADs corresponds to about 2 to 4 fold stabilization of LEF at DSB ends:

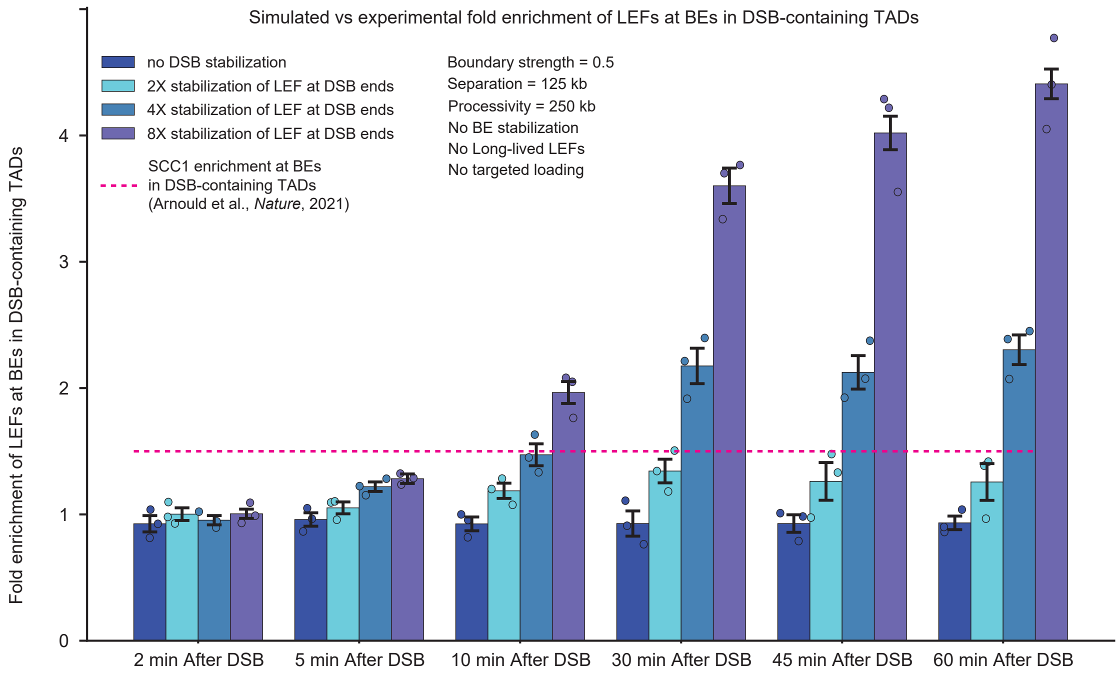

Simulated LEF enrichment at BEs in DSB-containing TADs is consistent with experimental observations. Fold enrichment is calculated as the number of LEFs in the DSB-containing TADs within 5 kb to the BEs at the indicated time points normalized against the number of LEFs in the same regions prior to DSB occurrence. The error bars represent standard error of mean, $n = 3$ independent 1D simulations, with the fold enrichment from each simulation overlaid as individual dots on the bar plot. The pink dashed line represents the SCC1 fold enrichment at BEs in the DSB-containing TAD determined by Arnould et al. [33].

Given the uncertainty associated with the experimental measurement, we use 8 as an upper bound for the fold increase in LEF lifetime due to stabilization by DSB ends, r:

$$r\epsilon[1, 8] \tag{161}$$

2.5 Estimation of the fold increase in LEF loading probability at DSB

Enrichment of cohesins at DSBs has been reported in several studies [33–36], and this enrichment was recently found to be dependent on cohesin loader NIPBL, as well as ATM and MRN complex recruited to DSB sites [33], pointing to a reactive mechanism that targets cohesin to DSB sites. We performed simulations with different fold increase in LEF loading probability at DSB, and found the ∼1.57 fold enrichment of SCC1 in the chromosome 20 DSB-containing TAD of DIva cells [33] corresponds to about 250 fold increase in LEF loading probability at DSB (**Supplementary Fig. 6a**). We implemented targeted loading by increasing the loading rate of LEFs within 1 kb of DSB for simplicity, whereas the observed accumulation of LEFs is not limited to the immediate proximity to DSBs but the whole DSB-containing TADs [33], suggesting LEFs might be targeted to larger regions around DSB instead

of just the DSB ends. Therefore, we compared the fold enrichment of LEFs in DSB-containing TADs here instead of just comparing the fold enrichment immediately around DSB ends. Given the noise in ChIP-seq experiments and the 2.5-5 times higher fold enrichment of SCC1 within 4kb around DSB reported by Cheblal et al. [36], we use 5000 (corresponding to 7 fold enrichment of LEFs in DSB-containing TADs) as an upper bound for the fold increase in LEF loading probability at DSB, F:

$$F\epsilon[1, 5000] \tag{162}$$

3 Supplementary Figures

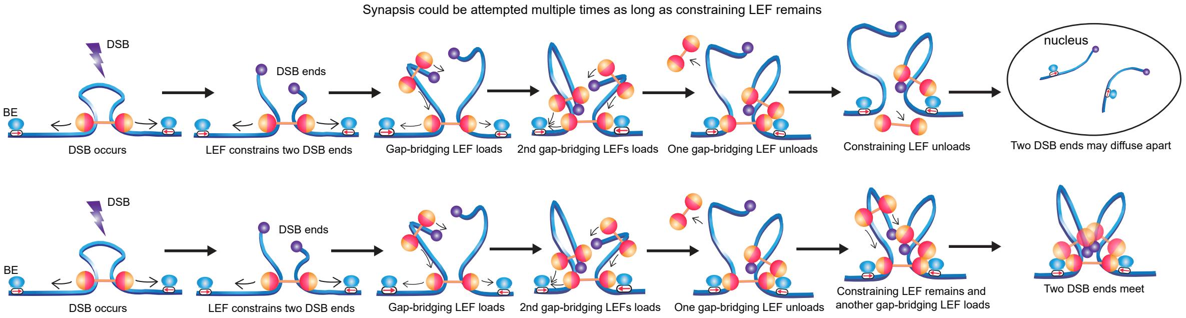

Supplementary Figure 1. **Synapsis could be attempted multiple times as long as constraining LEF remains.** Top row: we assume no further synapsis could be attempted once constraining LEF unloads as two DSB ends may diffuse apart. Bottom row: as long as constraining LEF remains, if gap-bridging LEFs unload before synapsis is achieved, additional gap-bridging LEFs could be loaded to attempt synapsis for multiple rounds until successful synapsis is achieved or the constraining LEF unloads.

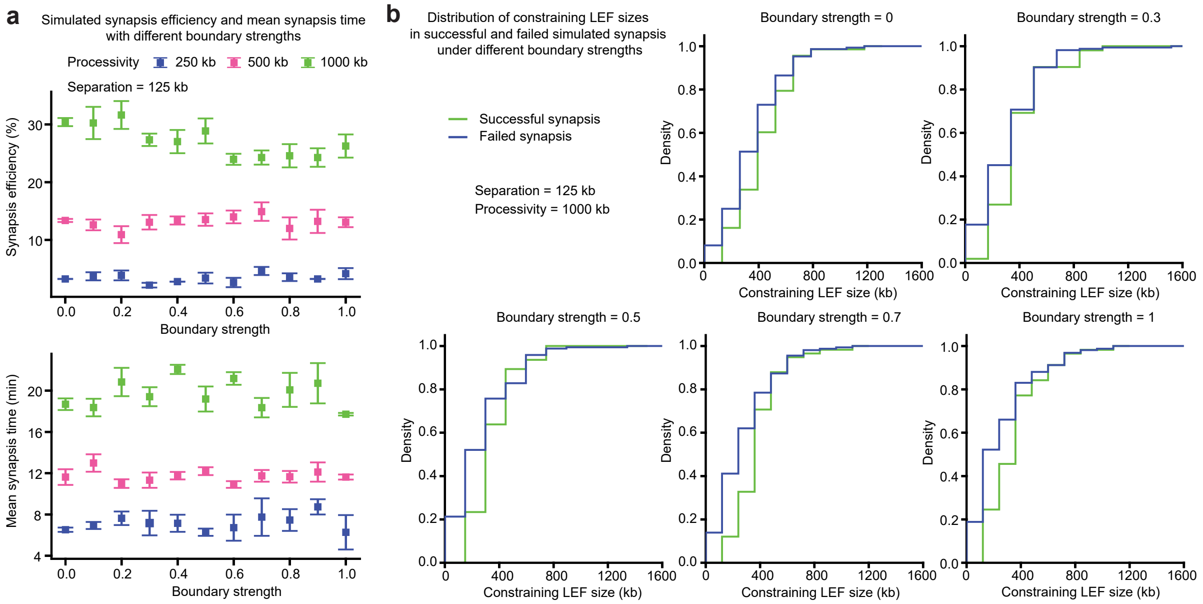

Supplementary Figure 2. **Effects of boundary strength on synapsis.** (**a**) Boundary strength has little impact on DSB end synapsis under the 3-parameter model. The error bars represent the standard error of mean, $n = 3$ independent 1D simulations. (**b**) Constraining LEF size does not correlate with boundary strength or whether synapsis succeeds or fails. The cumulative density function (CDF) of constraining LEF size in successful and failed simulated synapsis. Processivity = 250 kb, separation = 250 kb. Constraining LEF size was recorded at the moment when synapsis is achieved or at the moment when constraining LEF unloads for successful and failed synapsis events respectively.

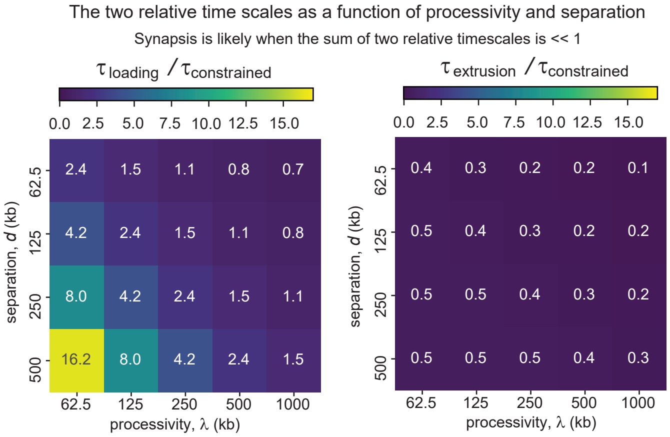

Supplementary Figure 3. **Loading of gap-bridging LEF is the rate limiting step of synapsis.** Heatmaps of $\tau_{\text{loading}}/\tau_{\text{constrained}}$ (left) and $\tau_{\text{extrusion}}/\tau_{\text{constrained}}$ (right) calculated from Eqs.(73)-(75) across different separation and processivity combinations.

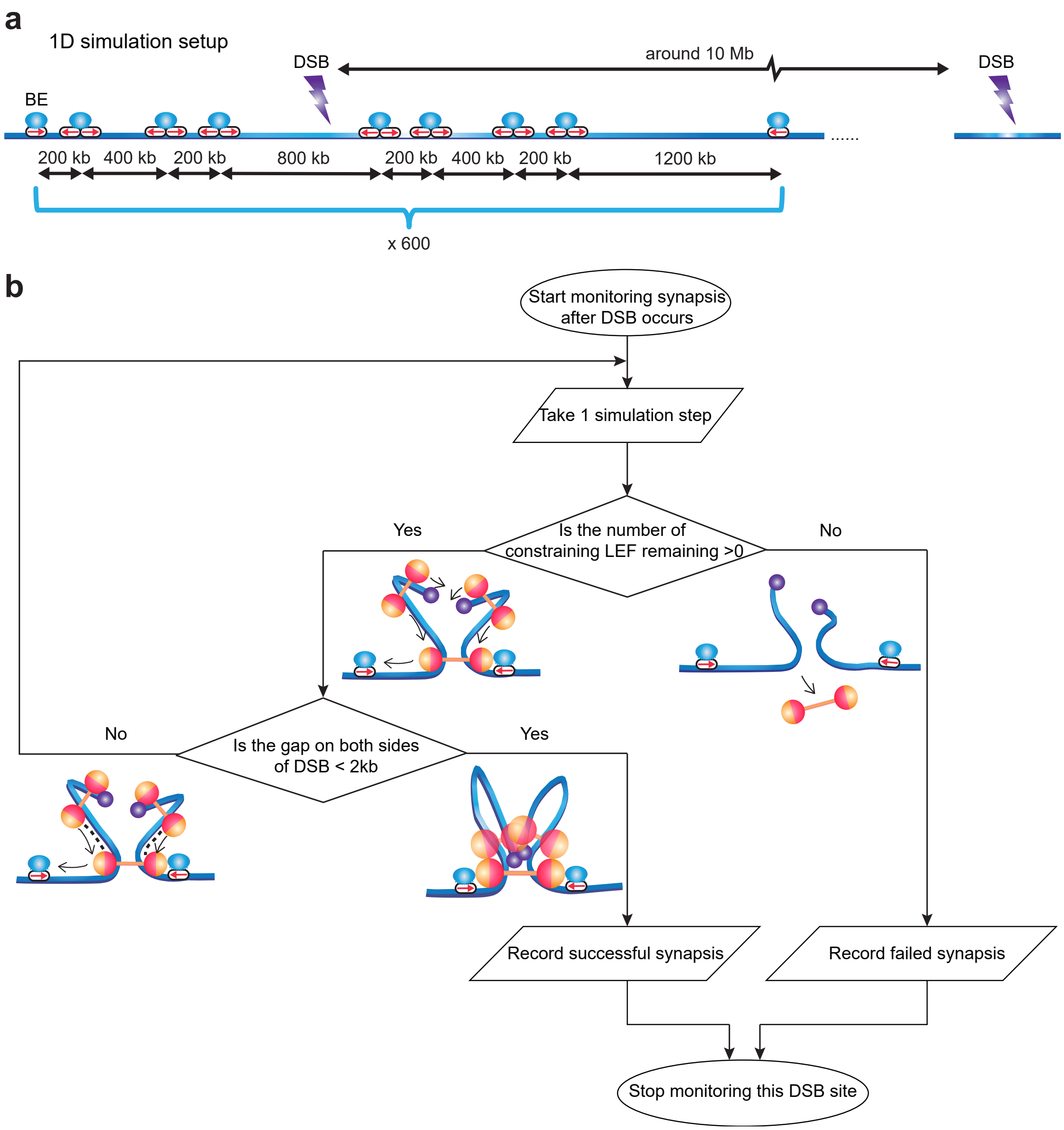

Supplementary Figure 4. **1D simulation setup and monitoring of synapsis event.** (**a**) Overview of 1D simulation setup. (**b**) After DSBs occur in 1D simulations, we start monitor synapsis event at individual DSB sites. At each simulation time step, we first checked if the there is at least one constraining LEF remaining for the DSB site. If there is no more constraining LEF at the DSB site, then we record a failed synapsis, and stop monitoring this DSB site in future simulation steps. If there is one or more constraining LEFs at the DSB site, then we go on to check the gap lengths on both sides of the DSB. If the gap on both sides of the DSB are smaller than 2kb, then we record a successful synapsis; otherwise we continue monitoring this DSB site in the future simulation steps until a successful synapsis or a failed synapsis is recorded.

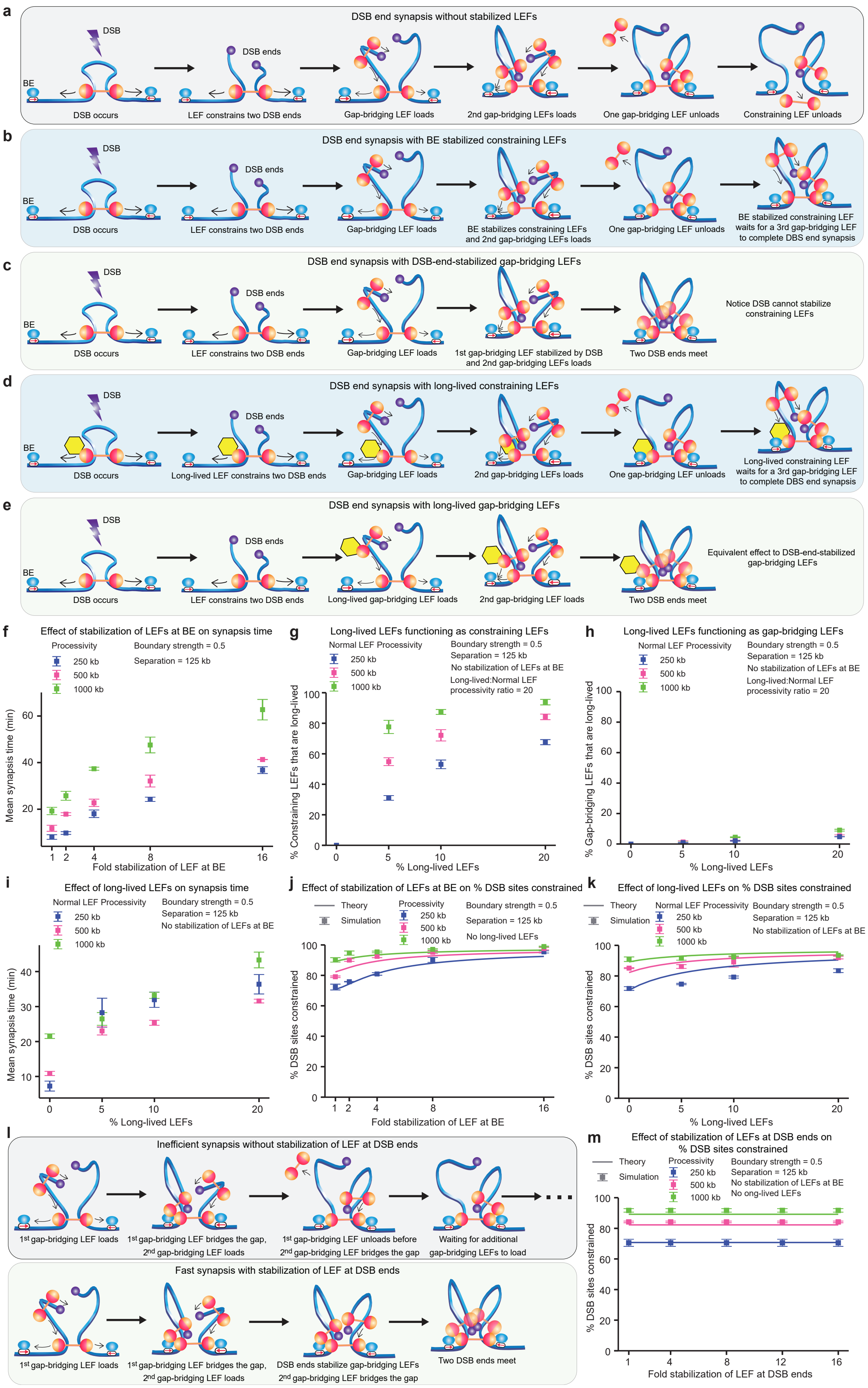
a
DSB end synapsis without stabilized LEFs
DSB
BE
DSB ends
DSB occurs
LEF constrains two DSB ends
Gap-bridging LEF loads
2nd gap-bridging LEFs loads
One gap-bridging LEF unloads
Constraining LEF unloads
b
DSB end synapsis with BE stabilized constraining LEFs
DSB occurs
LEF constrains two DSB ends
Gap-bridging LEF loads
BE stabilizes constraining LEFs and 2nd gap-bridging LEFs loads
One gap-bridging LEF unloads
BE stabilized constraining LEF waits for a 3rd gap-bridging LEF to complete DBS end synapsis
c
DSB end synapsis with DSB-end-stabilized gap-bridging LEFs
DSB occurs
LEF constrains two DSB ends
Gap-bridging LEF loads
1st gap-bridging LEF stabilized by DSB and 2nd gap-bridging LEFs loads
Two DSB ends meet
Notice DSB cannot stabilize constraining LEFs
d
DSB end synapsis with long-lived constraining LEFs
DSB occurs
Long-lived LEF constrains two DSB ends
Gap-bridging LEF loads
2nd gap-bridging LEFs loads
One gap-bridging LEF unloads
Long-lived constraining LEF waits for a 3rd gap-bridging LEF to complete DBS end synapsis
e
DSB end synapsis with long-lived gap-bridging LEFs
DSB occurs
LEF constrains two DSB ends
Long-lived gap-bridging LEF loads
2nd gap-bridging LEF loads
Two DSB ends meet
Equivalent effect to DSB-end-stabilized gap-bridging LEFs
f
Effect of stabilization of LEFs at BE on synapsis time
Processivity
250 kb
500 kb
1000 kb
Boundary strength = 0.5
Separation = 125 kb
Mean synapsis time (min)
Fold stabilization of LEF at BE
g
Long-lived LEFs functioning as constraining LEFs
Normal LEF Processivity
Boundary strength = 0.5
Separation = 125 kb
No stabilization of LEFs at BE
Long-lived:Normal LEF processivity ratio = 20
% Constraining LEFs that are long-lived
% Long-lived LEFs
h
Long-lived LEFs functioning as gap-bridging LEFs
% Gap-bridging LEFs that are long-lived
i
Effect of long-lived LEFs on synapsis time
j
Effect of stabilization of LEFs at BE on % DSB sites constrained
Theory
Simulation
No long-lived LEFs
% DSB sites constrained
k
Effect of long-lived LEFs on % DSB sites constrained
l
Inefficient synapsis without stabilization of LEF at DSB ends
1st gap-bridging LEF loads
1st gap-bridging LEF bridges the gap, 2nd gap-bridging LEF loads
1st gap-bridging LEF unloads before 2nd gap-bridging LEF bridges the gap
Waiting for additional gap-bridging LEFs to load
Fast synapsis with stabilization of LEF at DSB ends
DSB ends stabilize gap-bridging LEFs 2nd gap-bridging LEF bridges the gap
Two DSB ends meet
m
Effect of stabilization of LEFs at DSB ends on % DSB sites constrained
No ong-lived LEFs
Fold stabilization of LEF at DSB ends

Supplementary Figure 5. **Mechanisms of synapsis facilitated by stabilization of LEFs by BE and DSB, and a small portion of long-lived LEFs.** (**a**-**e**) Schematic diagrams of synapsis with LEFs without stabilization (a), with constraining LEFs stabilized by BE (b), with gap-bridging LEFs stabilized by DSB ends (c), with long-lived constraining LEFs (d), or with long-lived gap-bridging LEFs (e). (**f**) The effect of stabilization of LEF at BE on the mean synapsis time. Conversion of simulation time steps to synapsis time assumes total extrusion speed of 1 kb/s. The error bars represent the standard error of mean, n = 3 independent 1D simulations, with 216-218 DSB events per simulation. (**g**) Percentages of constraining LEFs that are long-lived. The error bars represent the standard error of mean, n = 3 independent 1D simulations, with 216-218 DSB events per simulation. (**h**) Percentages of gap-bridging LEFs that are long-lived. The error bars represent the standard error of mean, n = 3 independent 1D simulations, with 216-218 DSB events per simulation. (**i**) The effect of stabilization of LEF at BE on the mean synapsis time. Conversion of simulation time steps to synapsis time assumes total extrusion speed of 1 kb/s. The error bars represent the standard error of mean, n = 3 independent 1D simulations, with 216-218 DSB events per simulation. (**j,k**) The effect of stabilization of LEF at BE (j) and a small subpopulation of long-lived LEFs (k) on the probability of DSB occurring inside a DNA loop. The error bars represent the standard error of mean, n = 3 independent 1D simulations, with 216-218 DSB events per simulation. (**l**) Schematic diagram of synapsis with LEF stabilization by DSB ends, leading to more efficient synapsis. (**m**) DSB stabilization has no impact on the probability of DSB occurring inside a DNA loop. The error bars represent the standard error of mean, n = 3 independent 1D simulations, with 216-218 DSB events per simulation.

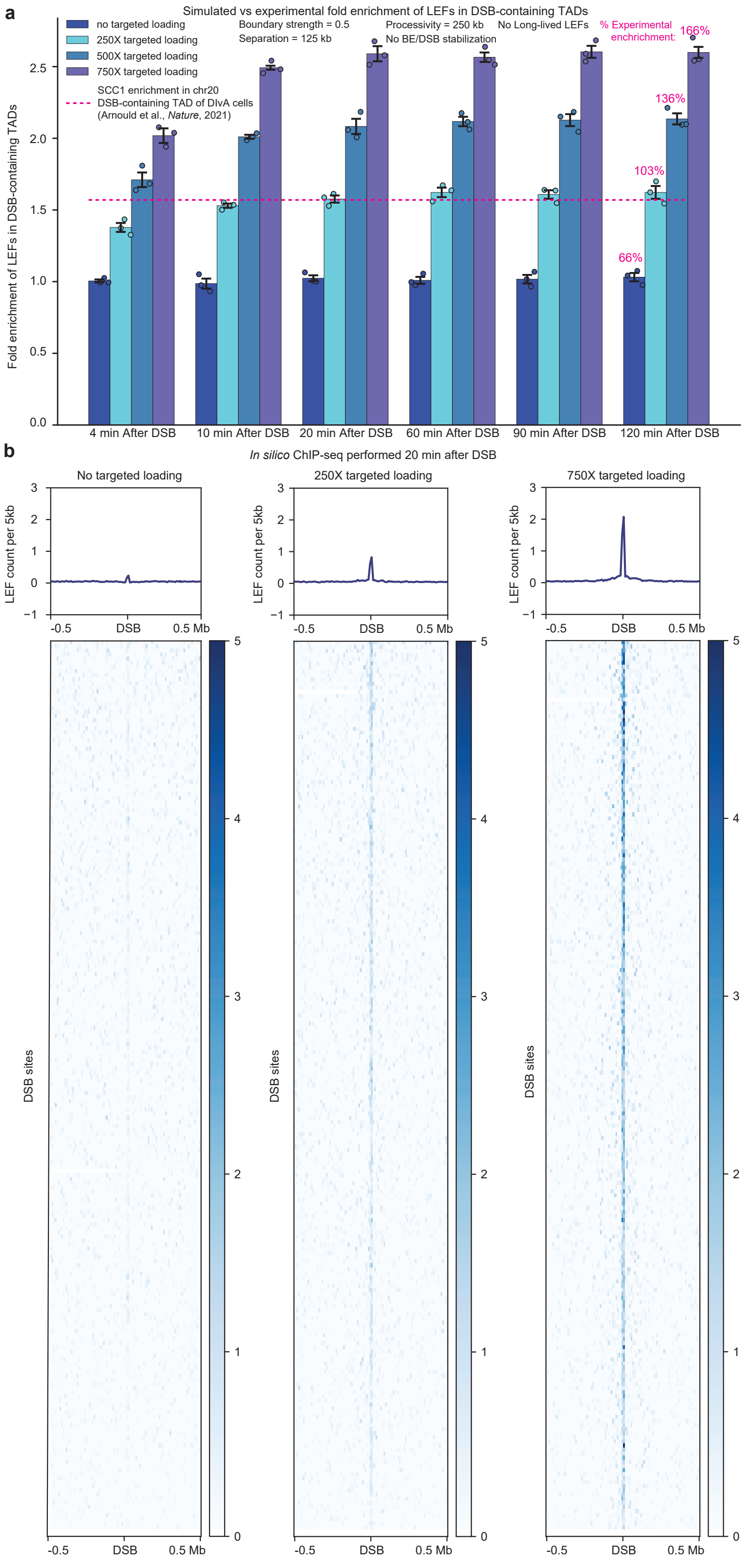

Supplementary Figure 6. ***In silico* ChIP-seq results showing LEF enrichment at DSB consistent with experimental observations.** (**a**) Fold enrichment of LEFs in DSB-containing TADs. Fold enrichment is calculated as the number of LEFs in the DSB-containing TADs at the indicated time points normalized against the number of LEFs in DSB-containing TADs prior to DSB occurrence. The error bars represent standard error of mean, $n = 3$ independent 1D simulations, with the fold enrichment from each simulation overlaid as individual dots on the bar plot. The pink dashed line represents the ChIP-seq SCC1 enrichment in the DSB-containing TAD (TAD boundaries determined by CTCF ChIP-seq data) on chromosome 20 of DIvA cells [33]. The pink text labels on top of the bars indicate % experimental enrichment for each targeted loading factor at steady state. (**b**) Enrichment heatmaps and metaplots of LEFs at DSB sites 20 minutes post DSB occurrence, aligned by their distance from the DSB site along the genome (Mb). Bin size=5 kb.

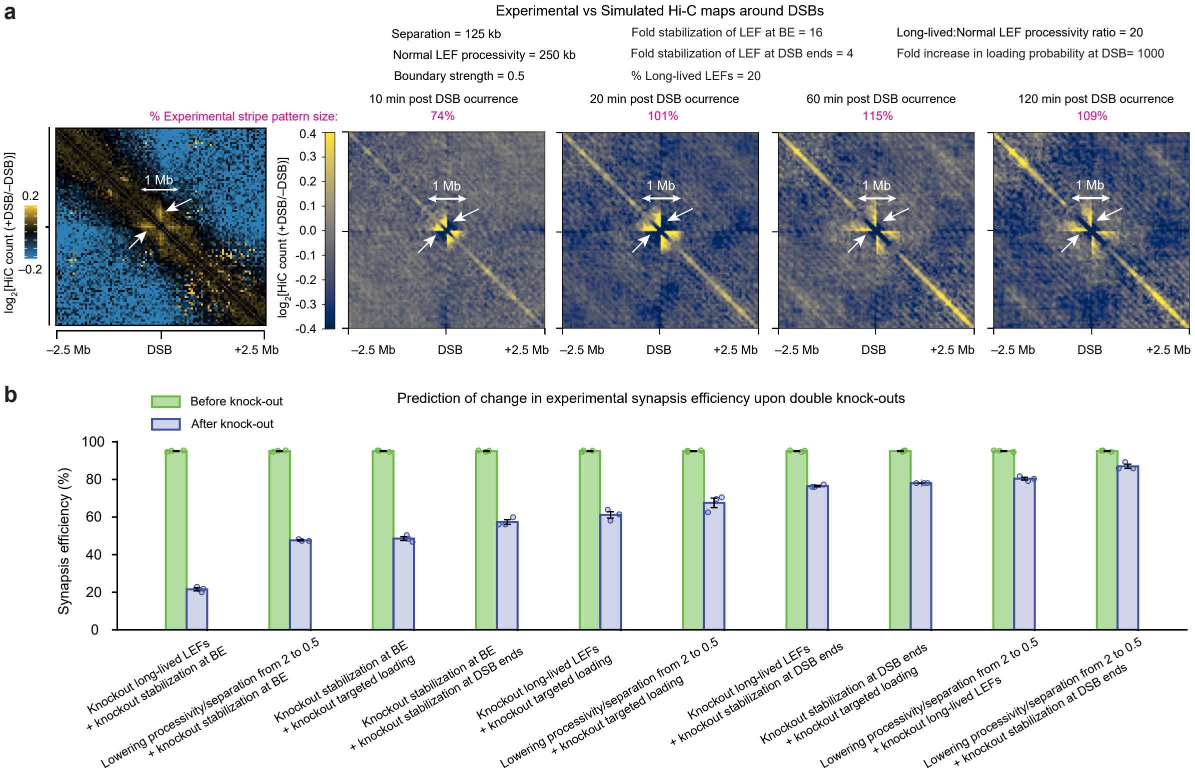

Supplementary Figure 7. **Parameter combination with $\geq$ 95% synapsis efficiency predicts Hi-C maps with similar pattern as experimental data, and double knock-outs significantly reduces synapsis efficiency for most knock-out combinations.** (**a**) The Hi-C contact maps of $\log_2$[+DSB/-DSB] centered on the DSBs at the indicated time points post DSB occurrence averaged over 603 DSBs (50-kb resolution, 5-Mb window) show a similar pattern to the experimental Hi-C map reused from Fig.2b by Arnould et al. [33] shown in the left. The contact maps are simulated with one parameter combination (indicated in legend) producing $\geq$ 95% synapsis efficiency. The white arrows highlight the stripe pattern. The stripe pattern grows quickly over time and reaches a steady state by 1 hr after DSB occurrence. Quantitative differences in contact frequency between our 1D simulations and the experiments may be due to differences in 1D simulation implementation versus experimental conditions (see **Methods**). The pink text labels on top of each simulated Hi-C map indicate % experimental stripe patter size over time.(**b**) The bar plot shows the synapsis efficiency before and after knocking out two of the five mechanisms discussed in **Fig. 6**. The error bars represent standard error of mean, n = 3 different parameter combinations that achieved $\geq$ 95% synapsis efficiency before knock-out, with the synapsis efficiency from each parameter combination overlaid as individual dots on the bar plot.

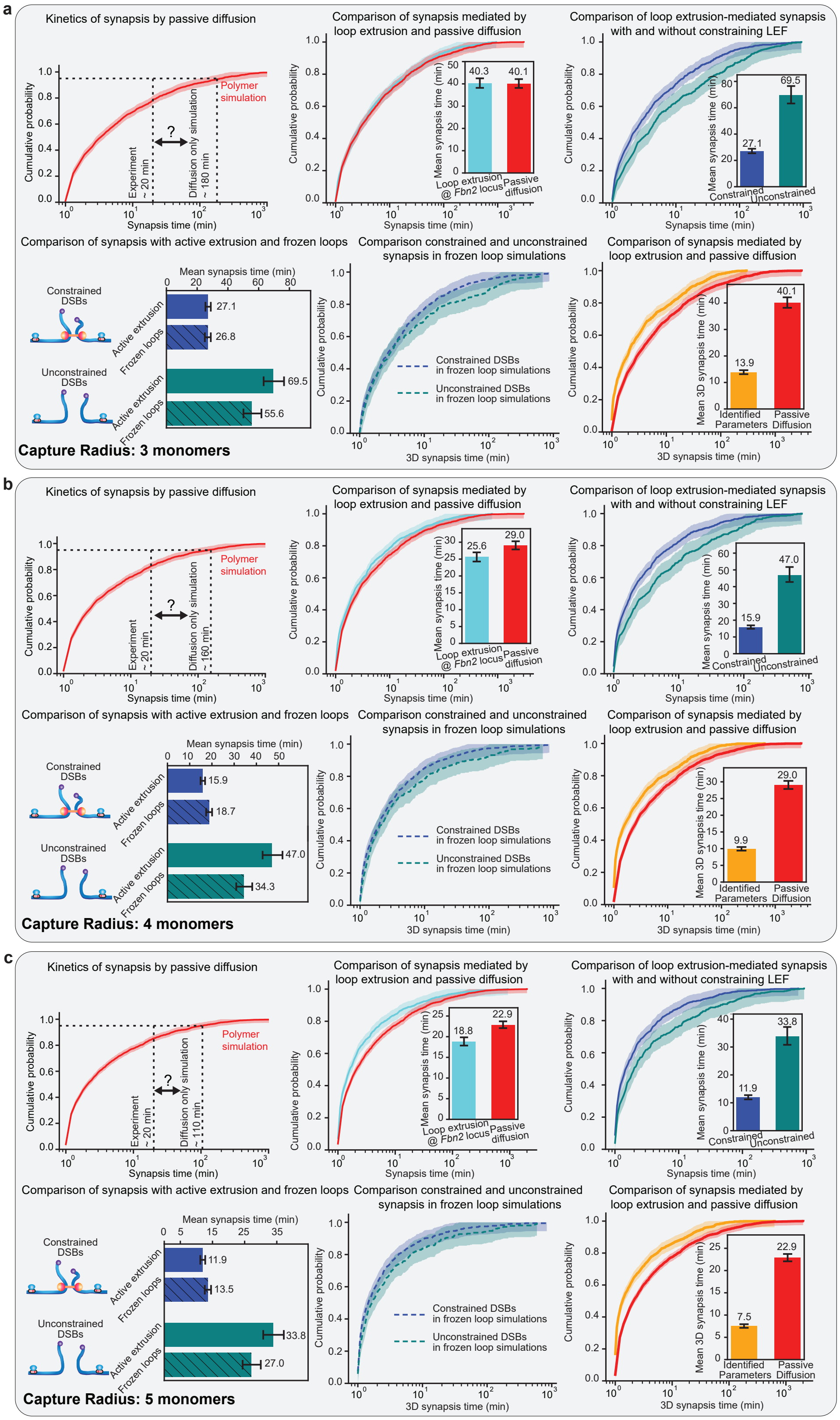

Supplementary Figure 8. **Comparison of 3D polymer simulation results across different capture radii.** (**a-c**) capture radius = 3 monomers (a), capture radius = 4 monomers (b, shown in the main text), capture radius = 5 monomers (c). Capture radius of 4 monomers was chosen given the average monomer displacement per time step, to avoid missing synapsis events. $n = 2333$ for simulated synapsis with passive diffusion alone; $n = 1376$ for simulated synapsis with loop extrusion at the *Fbn2* locus (among which 941 were at constrained DSBs and 435 were at unconstrained DSB); $n = 750$ for constrained DSBs and $n = 357$ for unconstrained DSBs in the frozen loop simulations; $n = 1313$ for simulated synapsis with loop extrusion parameters highlighted in main text **Fig. 5c**. The error bars in all bar plots represent 95% confidence interval of the mean using maximum likelihood estimation of the exponential distribution accounting for censored data [37].

Supplementary References

[1] Goloborodko, A., Marko, J. F. & Mirny, L. A. Chromosome compaction by active loop extrusion. *Biophysical journal* **110**, 2162–2168 (2016).

[2] Banigan, E. J. & Mirny, L. A. Limits of chromosome compaction by loop-extruding motors. *Physical Review X* **9**, 031007 (2019).

[3] Cattoglio, C. *et al.* Determining cellular CTCF and cohesin abundances to constrain 3D genome models. *Elife* **8**, e40164 (2019).

[4] Holzmann, J. *et al.* Absolute quantification of cohesin, CTCF and their regulators in human cells. *Elife* **8**, e46269 (2019).

[5] Lengronne, A. *et al.* Cohesin relocation from sites of chromosomal loading to places of convergent transcription. *Nature* **430**, 573–578 (2004).

[6] Newkirk, D. A. *et al.* The effect of nipped-B-like (Nipbl) haploinsufficiency on genome-wide cohesin binding and target gene expression: modeling Cornelia de Lange syndrome. *Clinical epigenetics* **9**, 1–20 (2017).

[7] Davidson, I. F. & Peters, J.-M. Genome folding through loop extrusion by SMC complexes. *Nature Reviews Molecular Cell Biology* **22**, 445–464 (2021).

[8] Busslinger, G. A. *et al.* Cohesin is positioned in mammalian genomes by transcription, CTCF and Wapl. *Nature* **544**, 503–507 (2017).

[9] Brandão, H. B. *et al.* RNA polymerases as moving barriers to condensin loop extrusion. *Proceedings of the National Academy of Sciences* **116**, 20489–20499 (2019).

[10] Canela, A. *et al.* Genome organization drives chromosome fragility. *Cell* **170**, 507–521 (2017).

[11] Shastri, N. *et al.* Genome-wide identification of structure-forming repeats as principal sites of fork collapse upon ATR inhibition. *Molecular cell* **72**, 222–238 (2018).

[12] Canela, A. *et al.* Topoisomerase II-induced chromosome breakage and translocation is determined by chromosome architecture and transcriptional activity. *Molecular cell* **75**, 252–266 (2019).

[13] Gothe, H. J. *et al.* Spatial chromosome folding and active transcription drive DNA fragility and formation of oncogenic MLL translocations. *Molecular cell* **75**, 267–283 (2019).

[14] Madabhushi, R. *et al.* Activity-induced DNA breaks govern the expression of neuronal early-response genes. *Cell* **161**, 1592–1605 (2015).

[15] Puerto, S. *et al.* Induction, processing and persistence of radiation-induced chromosomal aberrations involving hamster euchromatin and heterochromatin. *Mutation Research/Genetic Toxicology and Environmental Mutagenesis* **469**, 169–179 (2000).

[16] Hansen, A. S. CTCF as a boundary factor for cohesin-mediated loop extrusion: evidence for a multi-step mechanism. *Nucleus* **11**, 132–148 (2020).

[17] Ganji, M. *et al.* Real-time imaging of DNA loop extrusion by condensin. *Science* **360**, 102–105 (2018).

[18] Kong, M. *et al.* Human condensin i and ii drive extensive ATP-dependent compaction of nucleosome-bound DNA. *Molecular cell* **79**, 99–114 (2020).

[19] Golfier, S., Quail, T., Kimura, H. & Brugués, J. Cohesin and condensin extrude DNA loops in a cell cycle-dependent manner. *Elife* **9**, e53885 (2020).

[20] Kim, Y., Shi, Z., Zhang, H., Finkelstein, I. J. & Yu, H. Human cohesin compacts DNA by loop extrusion. *Science* **366**, 1345–1349 (2019).

[21] Davidson, I. F. *et al.* DNA loop extrusion by human cohesin. *Science* **366**, 1338–1345 (2019).

[22] Rao, S. S. *et al.* Cohesin loss eliminates all loop domains. *Cell* **171**, 305–320 (2017).

[23] Gibcus, J. H. *et al.* A pathway for mitotic chromosome formation. *Science* **359** (2018).

[24] Hansen, A. S., Pustova, I., Cattoglio, C., Tjian, R. & Darzacq, X. CTCF and cohesin regulate chromatin loop stability with distinct dynamics. *Elife* **6**, e25776 (2017).

[25] Walther, N. *et al.* A quantitative map of human Condensins provides new insights into mitotic chromosome architecture. *Journal of Cell Biology* **217**, 2309–2328 (2018).

[26] Li, Y. *et al.* The structural basis for cohesin–CTCF-anchored loops. *Nature* **578**, 472–476 (2020).

[27] Wutz, G. *et al.* ESCO1 and CTCF enable formation of long chromatin loops by protecting cohesinSTAG1 from WAPL. *Elife* **9**, e52091 (2020).

[28] Arruda, N. L. *et al.* Distinct and overlapping roles of STAG1 and STAG2 in cohesin localization and gene expression in embryonic stem cells. *Epigenetics & chromatin* **13**, 1–17 (2020).

[29] Gerlich, D., Koch, B., Dupeux, F., Peters, J.-M. & Ellenberg, J. Live-cell imaging reveals a stable cohesin-chromatin interaction after but not before DNA replication. *Current Biology* **16**, 1571–1578 (2006).

[30] Cuadrado, A. & Losada, A. Specialized functions of cohesins STAG1 and STAG2 in 3D genome architecture. *Current opinion in genetics & development* **61**, 9–16 (2020).

[31] Remeseiro, S., Cuadrado, A., Gómez-López, G., Pisano, D. G. & Losada, A. A unique role of cohesin-SA1 in gene regulation and development. *The EMBO journal* **31**, 2090–2102 (2012).

[32] Kojic, A. *et al.* Distinct roles of cohesin-SA1 and cohesin-SA2 in 3D chromosome organization. *Nature structural & molecular biology* **25**, 496–504 (2018).

[33] Arnould, C. *et al.* Loop extrusion as a mechanism for formation of DNA damage repair foci. *Nature* **590**, 660–665 (2021).

[34] Caron, P. *et al.* Cohesin protects genes against γH2AX induced by DNA double-strand breaks. *PLoS genetics* **8**, e1002460 (2012).

[35] Meisenberg, C. *et al.* Repression of transcription at DNA breaks requires cohesin throughout interphase and prevents genome instability. *Molecular cell* **73**, 212–223 (2019).

[36] Cheblal, A. *et al.* DNA damage-induced nucleosome depletion enhances homology search independently of local break movement. *Molecular Cell* **80**, 311–326 (2020).

[37] Gabriele, M. *et al.* Dynamics of CTCF- and cohesin-mediated chromatin looping revealed by live-cell imaging. *Science* **376**, 496–501 (2022).
