# Supplementary material for: DNA double-strand break end synapsis by DNA loop extrusion
Source: Nat Commun. 2023 Apr 6;14:1913. doi: 10.1038/s41467-023-37583-w (PMC10079674; doi:10.1038/s41467-023-37583-w)
Supplement: Supplementary file 3 — Description of Additional Supplementary Files [file 41467_2023_37583_MOESM3_ESM.pdf]

Description of Additional Supplementary Files

File Name: Supplementary Movie 1
Description: Example of a successful synapsis event in a 1D simulation. Simulation parameters: separation = 125 kb, processivity = 1000 kb, boundary strength = 0.5, and no mechanistic extensions

File Name: Supplementary Movie 2
Description: Example of a failed synapsis event in a 1D simulation, simulated with the simple 3-parameter loop extrusion model. Simulation parameters: separation = 125 kb, processivity = 1000 kb, boundary strength = 0.5, and no mechanistic extensions.

File Name: Supplementary Movie 3
Description: Example video of synapsis with stabilization of LEF at BE in a 1D simulation. Simulation parameters: separation = 125 kb, processivity = 250 kb, boundary strength = 0.5, fold stabilization of LEF at BE = 16, and no other mechanistic extensions.

File Name: Supplementary Movie 4
Description: Example video of synapsis with a small fraction of long-lived LEFs in a 1D simulation. Simulation parameters: separation = 125 kb, normal LEF processivity = 250 kb, boundary strength = 0.5, % long-lived LEFs = 20, long-lived:normal LEF processivity ratio = 20, and no other mechanistic extensions.

File Name: Supplementary Movie 5
Description: Example video of synapsis with stabilization of LEF at DSB ends in a 1D simulation. Simulation parameters: separation = 125 kb, processivity = 250 kb, boundary strength = 0.5, fold increase in loading probability at DSB = 1000, and no other mechanistic extensions.

File Name: Supplementary Movie 6
Description: Example video of synapsis with targeted loading of LEFs at DSB in a 1D simulation. Simulation parameters: separation = 125 kb, processivity = 250 kb, boundary strength = 0.5, fold stabilization of LEF at DSB ends = 4, and no other mechanistic extensions.

File Name: Supplementary Movie 7
Description: Example video of synapsis with all four mechanistic extensions combined in a 1D simulation. Simulation parameters: separation = 125 kb, normal LEF processivity = 250 kb, boundary strength = 0.5, fold stabilization of LEF at BE = 16, % long-lived LEFs = 20, long-lived:normal LEF processivity ratio = 20, fold stabilization of LEF at DSB ends = 4, and fold increase in loading probability at DSB = 1000.
