# Supplementary material for: DNA double-strand break end synapsis by DNA loop extrusion
Source: Nat Commun. 2023 Apr 6;14:1913. doi: 10.1038/s41467-023-37583-w (PMC10079674; doi:10.1038/s41467-023-37583-w)
Supplement: Supplementary file 11 — Reporting Summary [file 41467_2023_37583_MOESM11_ESM.pdf]

nature portfolio

Corresponding author(s): Anders S. Hansen, Hugo B. Brandão

Last updated by author(s): Feb 26, 2023

Reporting Summary

Nature Portfolio wishes to improve the reproducibility of the work that we publish. This form provides structure for consistency and transparency in reporting. For further information on Nature Portfolio policies, see our Editorial Policies and the Editorial Policy Checklist.

Statistics

For all statistical analyses, confirm that the following items are present in the figure legend, table legend, main text, or Methods section.

n/a	Confirmed	
☐	☒	The exact sample size (*n*) for each experimental group/condition, given as a discrete number and unit of measurement
☐	☒	A statement on whether measurements were taken from distinct samples or whether the same sample was measured repeatedly
☐	☒	The statistical test(s) used AND whether they are one- or two-sided<br>*Only common tests should be described solely by name; describe more complex techniques in the Methods section.*
☒	☐	A description of all covariates tested
☒	☐	A description of any assumptions or corrections, such as tests of normality and adjustment for multiple comparisons
☐	☒	A full description of the statistical parameters including central tendency (e.g. means) or other basic estimates (e.g. regression coefficient) AND variation (e.g. standard deviation) or associated estimates of uncertainty (e.g. confidence intervals)
☐	☒	For null hypothesis testing, the test statistic (e.g. *F*, *t*, *r*) with confidence intervals, effect sizes, degrees of freedom and *P* value noted<br>*Give P values as exact values whenever suitable.*
☒	☐	For Bayesian analysis, information on the choice of priors and Markov chain Monte Carlo settings
☒	☐	For hierarchical and complex designs, identification of the appropriate level for tests and full reporting of outcomes
☒	☐	Estimates of effect sizes (e.g. Cohen's *d*, Pearson's *r*), indicating how they were calculated

Our web collection on statistics for biologists contains articles on many of the points above.

Software and code

Policy information about availability of computer code

Data collection	Source codes for running simulations and data collection are available at: https://github.com/ahansenlab/DNA_break_synapsis_models. Polychrom (v0.1.0) that wraps OpenMM(v7.6.0) was used to run 3D polymer simulations.
Data analysis	Source codes for analysis of simulation results and for generating ChIP-seq and Hi-C simulations are deposited at: https://github.com/ahansenlab/DNA_break_synapsis_models. deepTools(v3.5.0) was used to generate ChIP-seq heatmaps.

For manuscripts utilizing custom algorithms or software that are central to the research but not yet described in published literature, software must be made available to editors and reviewers. We strongly encourage code deposition in a community repository (e.g. GitHub). See the Nature Portfolio guidelines for submitting code & software for further information.

Data

Policy information about availability of data

All manuscripts must include a data availability statement. This statement should provide the following information, where applicable:

- Accession codes, unique identifiers, or web links for publicly available datasets
- A description of any restrictions on data availability
- For clinical datasets or third party data, please ensure that the statement adheres to our policy

Simulation data are deposited at: https://github.com/ahansenlab/DNA_break_synapsis_models/tree/master/Data. Analytical theory formulation is available at: https://github.com/ahansenlab/DNA_break_synapsis_models/tree/master/Mathematica%20Notebooks.

Human research participants

Policy information about studies involving human research participants and Sex and Gender in Research.

Reporting on sex and gender	N/A
Population characteristics	N/A
Recruitment	N/A
Ethics oversight	N/A

Note that full information on the approval of the study protocol must also be provided in the manuscript.

Field-specific reporting

Please select the one below that is the best fit for your research. If you are not sure, read the appropriate sections before making your selection.

☒ Life sciences ☐ Behavioural & social sciences ☐ Ecological, evolutionary & environmental sciences

For a reference copy of the document with all sections, see nature.com/documents/nr-reporting-summary-flat.pdf

Life sciences study design

All studies must disclose on these points even when the disclosure is negative.

Sample size	Each 1D simulation run had 216-218 DSB events, from which synapsis efficiency and mean synapsis time were calculated; each 3D polymer simulation condition had more than 1100 DSB events (exact values specified in figure legends), from which the CDF of synapsis time and mean synapsis time were calculated. No statistical methods were used to estimate sample size. Sample size was determined based on prior studies in the field to generate reproducible results.
Data exclusions	No data were excluded.
Replication	Three independent runs of 1D simulations were performed for each data point; at least fifty independent runs of 3D polymer simulations were performed for each CDF curve.<br><br>All attempts for replication were successful.
Randomization	The DSBs were randomly introduced within TADs in all simulations.
Blinding	Binding was not relevant due to the design of our study. Observer bias was prevented because the simulations were performed using automated python and cython scripts and the analysis was performed using automated python script.

Reporting for specific materials, systems and methods

We require information from authors about some types of materials, experimental systems and methods used in many studies. Here, indicate whether each material, system or method listed is relevant to your study. If you are not sure if a list item applies to your research, read the appropriate section before selecting a response.

Materials & experimental systems

n/a	Involved in the study	
☒	☐	Antibodies
☒	☐	Eukaryotic cell lines
☒	☐	Palaeontology and archaeology
☒	☐	Animals and other organisms
☒	☐	Clinical data
☒	☐	Dual use research of concern

Methods

n/a	Involved in the study	
☒	☐	ChIP-seq
☒	☐	Flow cytometry
☒	☐	MRI-based neuroimaging
